# Supplementary material for: Antibacterial, Antifungal and Anticancer Activities of Compounds Produced by Newly Isolated Streptomyces Strains from the Szczelina Chochołowska Cave (Tatra Mountains, Poland)
Source: Antibiotics (Basel). 2021 Oct 5;10(10):1212. doi: 10.3390/antibiotics10101212 (PMC8532742; doi:10.3390/antibiotics10101212)
Supplement: Supplementary file 1 [file antibiotics-10-01212-s001.zip › antibiotics-1416584-supplementary.pdf]

Candida parapsilosis D2 (Bruss Laboratories, Gdynia, Poland)<br><br>*Candida glabrata* D3 (Bruss Laboratories, Gdynia, Poland)<br><br>*Candida tropicalis* D4 (Bruss Laboratories, Gdynia, Poland)	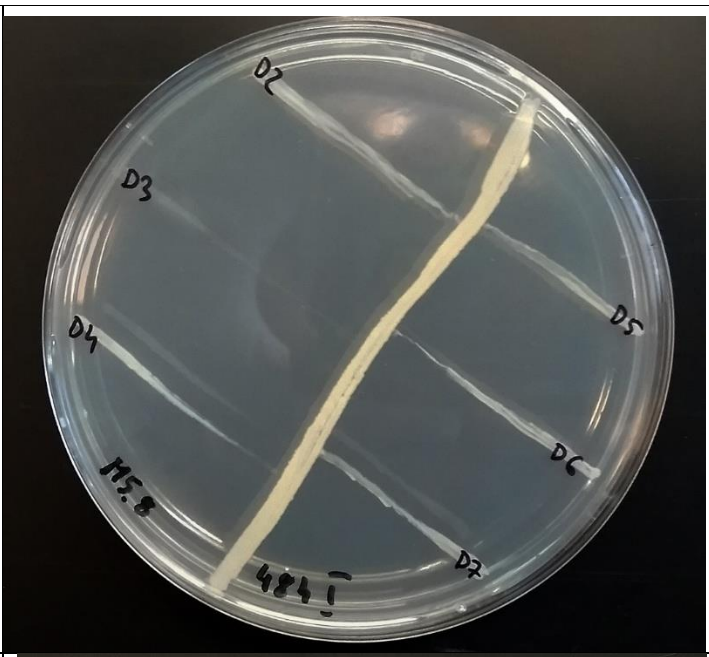 M5_8 48h I	*Candida dubliniensis* D5 (Bruss Laboratories, Gdynia, Poland)<br><br>*Candida albicans* D6 (Bruss Laboratories, Gdynia, Poland)<br><br>*Candida albicans* D7 (Medical University of Gdańsk)
Candida albicans D8 (Medical University of Gdańsk)<br><br>*Candida albicans* D9 (University Clinical Centre in Gdańsk)<br><br>*Candida albicans* E1 (University Clinical Centre in Gdańsk)	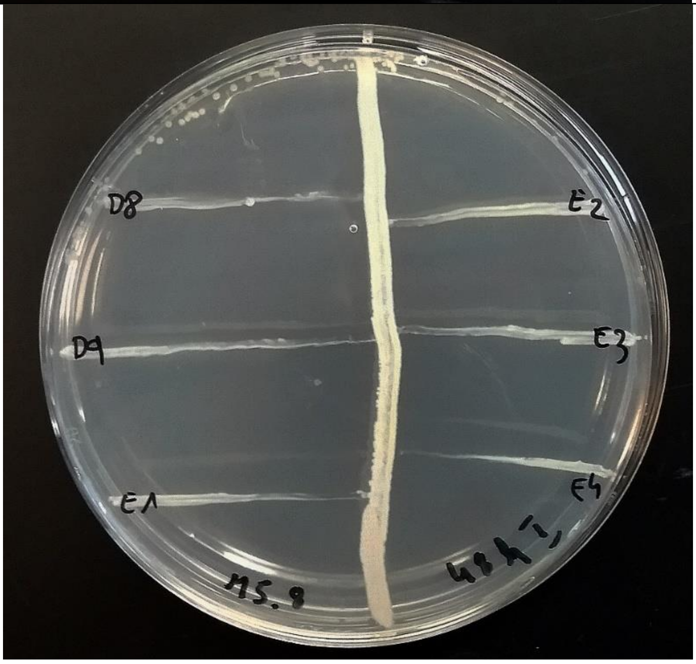<br>M5_8 48h I	*Candida guilliermondii* E2 (University Clinical Centre in Gdańsk)<br><br>*Candida guilliermondii* E3 (University Clinical Centre in Gdańsk)<br><br>*Candida albicans* E4 (University Clinical Centre in Gdańsk)
Candida albicans E5 (University Clinical Centre in Gdańsk)<br><br>*Candida glabrata* E6 (University Clinical Centre in Gdańsk)<br><br>*Candida glabrata* E7 (University Clinical Centre in Gdańsk)	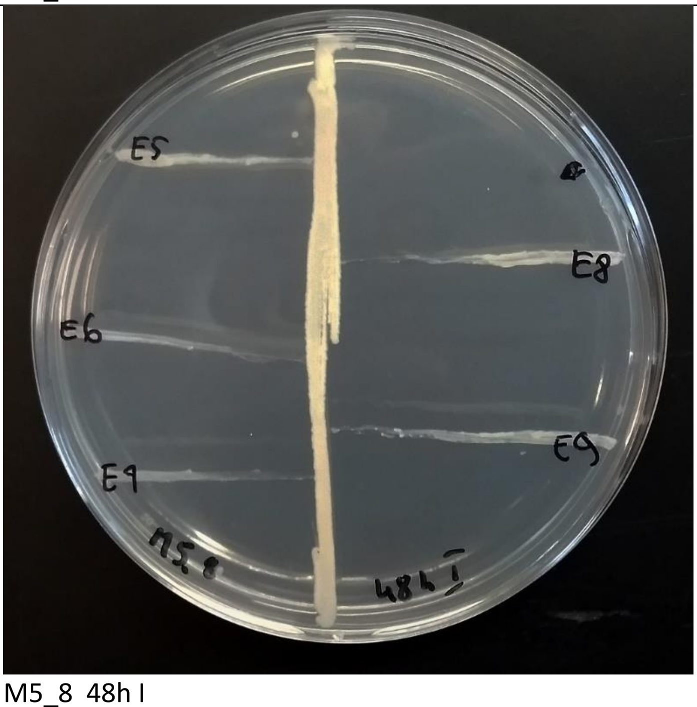<br>M5_8 48h I	*Candida* sp. E8 (University Clinical Centre in Gdańsk)<br><br>*Candida* sp. E9 (University Clinical Centre in Gdańsk)

Candida parapsilosis D2 (Bruss Laboratories, Gdynia, Poland)<br><br>*Candida glabrata* D3 (Bruss Laboratories, Gdynia, Poland)<br><br>*Candida tropicalis* D4 (Bruss Laboratories, Gdynia, Poland)	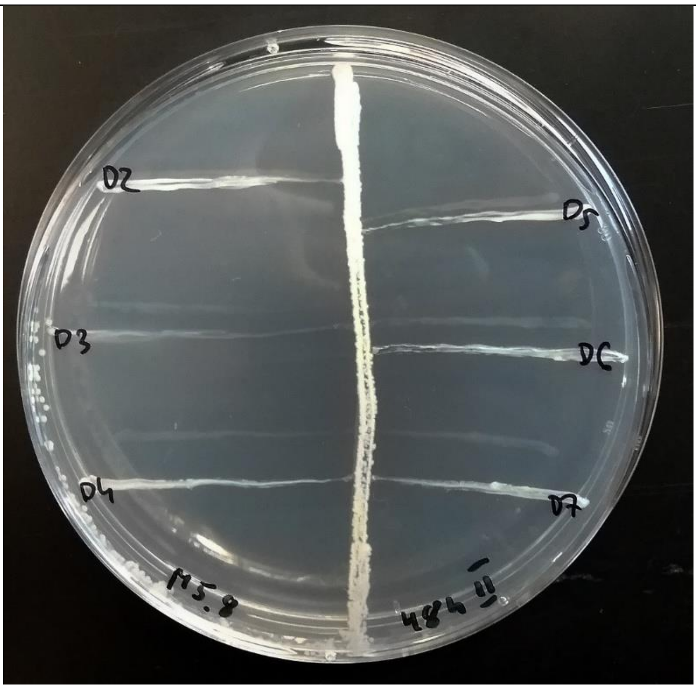<br>M5_8 48h II	*Candida dubliniensis* D5 (Bruss Laboratories, Gdynia, Poland)<br><br>*Candida albicans* D6 (Bruss Laboratories, Gdynia, Poland)<br><br>*Candida albicans* D7 (Medical University of Gdańsk)
Candida albicans D8 (Medical University of Gdańsk)<br><br>*Candida albicans* D9 (University Clinical Centre in Gdańsk)<br><br>*Candida albicans* E1 (University Clinical Centre in Gdańsk)	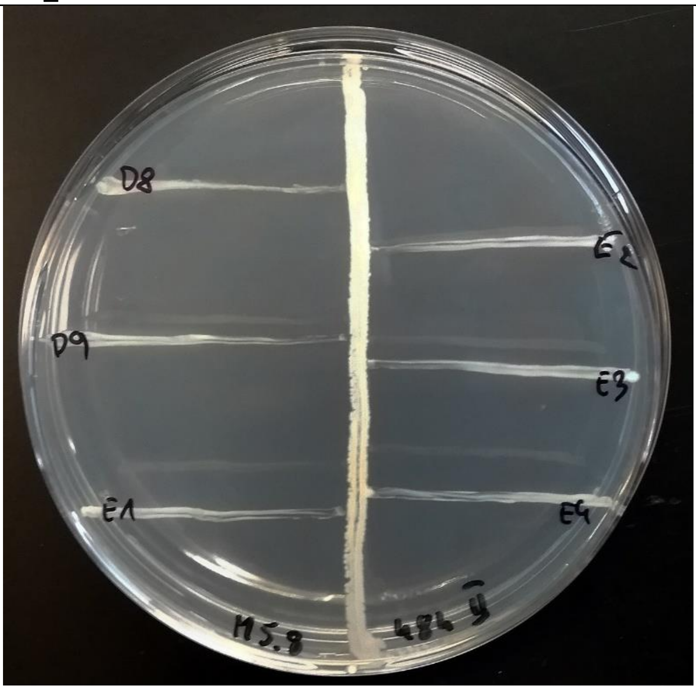<br>M5_8 48h II	*Candida guilliermondii* E2 (University Clinical Centre in Gdańsk)<br><br>*Candida guilliermondii* E3 (University Clinical Centre in Gdańsk)<br><br>*Candida albicans* E4 (University Clinical Centre in Gdańsk)

<table>
<tr>
<td>Candida albicans E5 (University Clinical Centre in Gdańsk)

Candida glabrata E6 (University Clinical Centre in Gdańsk)

Candida glabrata E7 (University Clinical Centre in Gdańsk)</td>
<td>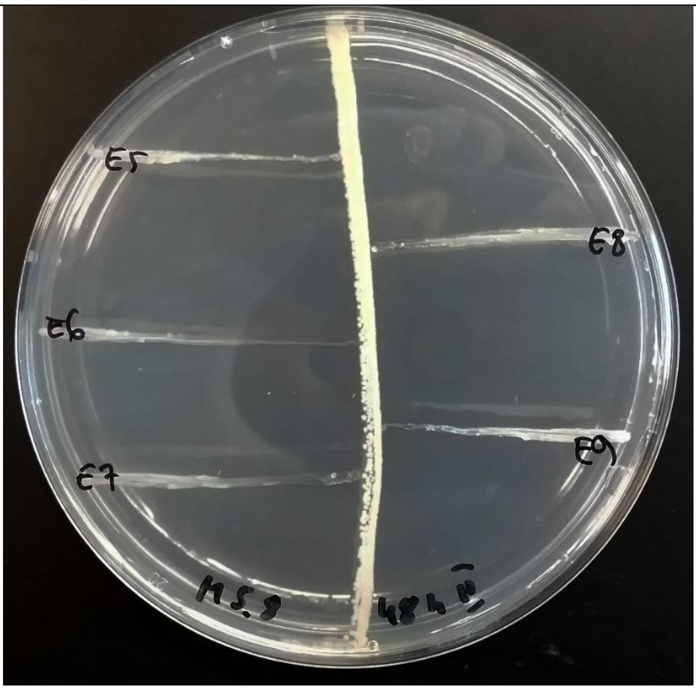

M5_8 48h II</td>
<td>Candida sp. E8 (University Clinical Centre in Gdańsk)

Candida sp. E9 (University Clinical Centre in Gdańsk)</td>
</tr>
<tr>
<td>Candida parapsilosis D2 (Bruss Laboratories, Gdynia, Poland)

Candida glabrata D3 (Bruss Laboratories, Gdynia, Poland)

Candida tropicalis D4 (Bruss Laboratories, Gdynia, Poland)</td>
<td>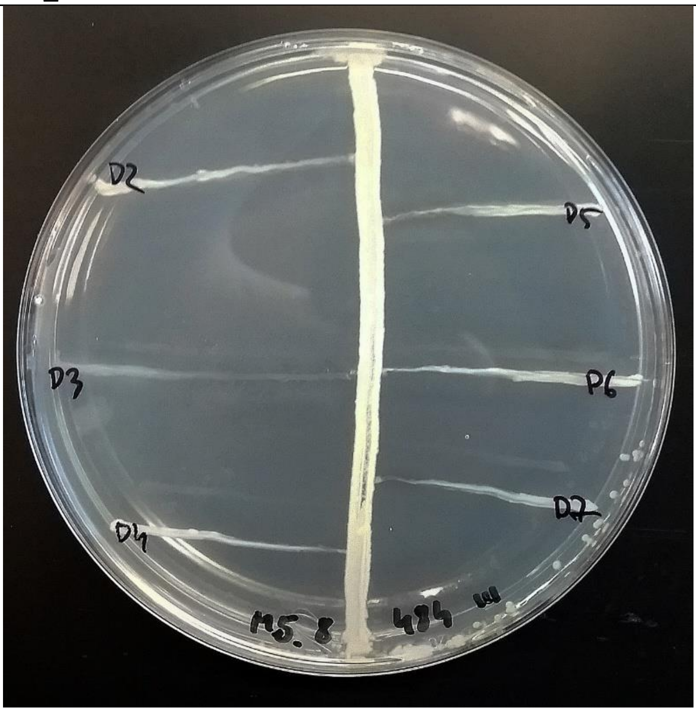

M5_8 48h III</td>
<td>Candida dubliniensis D5 (Bruss Laboratories, Gdynia, Poland)

Candida albicans D6 (Bruss Laboratories, Gdynia, Poland)

Candida albicans D7 (Medical University of Gdańsk)</td>
</tr>
</table>

Candida albicans D8 (Medical University of Gdańsk)<br><br>*Candida albicans* D9 (University Clinical Centre in Gdańsk)<br><br>*Candida albicans* E1 (University Clinical Centre in Gdańsk)	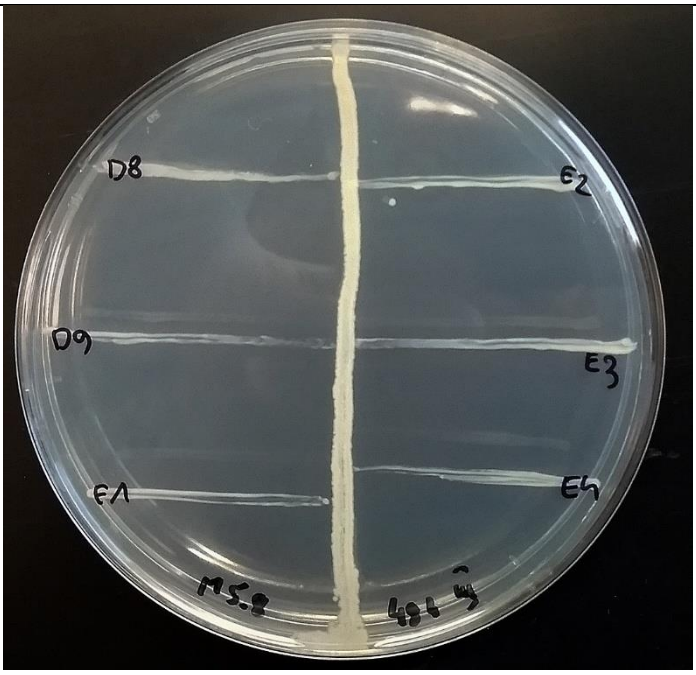<br>M5_8 48h III	*Candida guilliermondii* E2 (University Clinical Centre in Gdańsk)<br><br>*Candida guilliermondii* E3 (University Clinical Centre in Gdańsk)<br><br>*Candida albicans* E4 (University Clinical Centre in Gdańsk)
Candida albicans E5 (University Clinical Centre in Gdańsk)<br><br>*Candida glabrata* E6 (University Clinical Centre in Gdańsk)<br><br>*Candida glabrata* E7 (University Clinical Centre in Gdańsk)	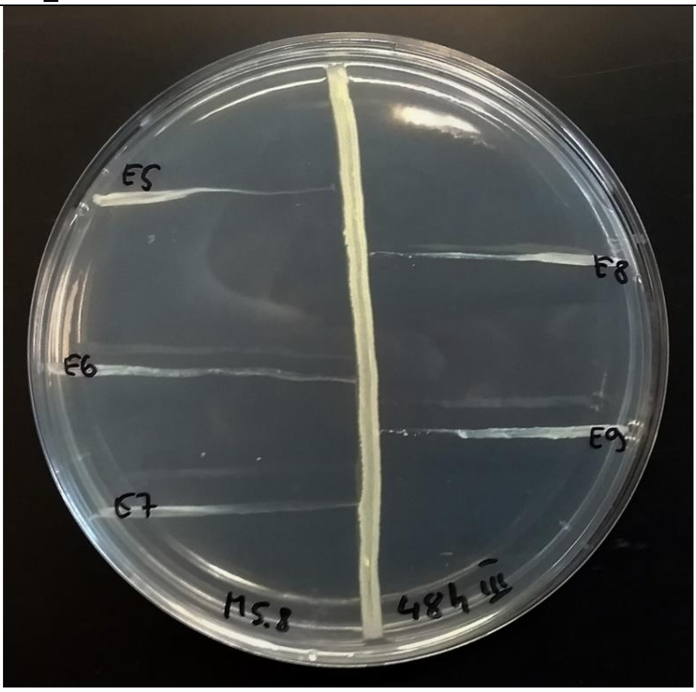<br>M5_8 48h III	*Candida* sp. E8 (University Clinical Centre in Gdańsk)<br><br>*Candida* sp. E9 (University Clinical Centre in Gdańsk)

Candida parapsilosis D2 (Bruss Laboratories, Gdynia, Poland)<br><br>*Candida glabrata* D3 (Bruss Laboratories, Gdynia, Poland)<br><br>*Candida tropicalis* D4 (Bruss Laboratories, Gdynia, Poland)	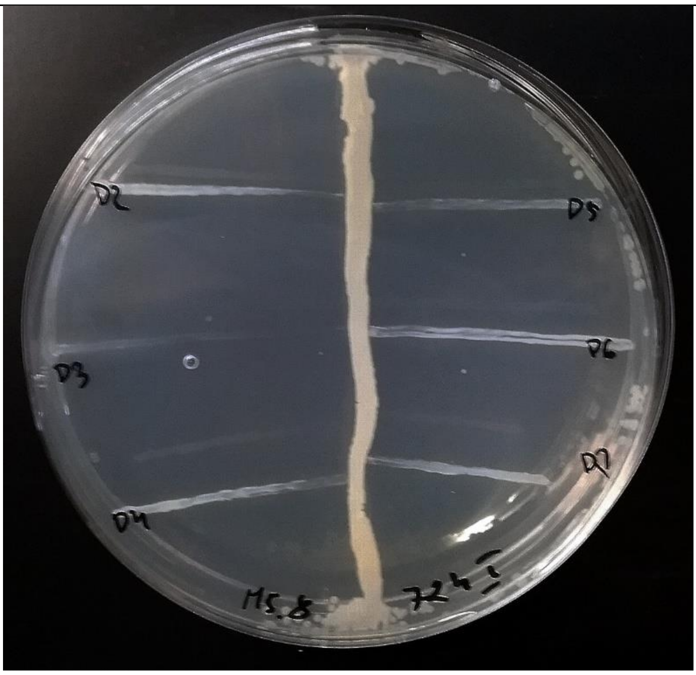<br>M5_8 72h I	*Candida dubliniensis* D5 (Bruss Laboratories, Gdynia, Poland)<br><br>*Candida albicans* D6 (Bruss Laboratories, Gdynia, Poland)<br><br>*Candida albicans* D7 (Medical University of Gdańsk)
Candida albicans D8 (Medical University of Gdańsk)<br><br>*Candida albicans* D9 (University Clinical Centre in Gdańsk)<br><br>*Candida albicans* E1 (University Clinical Centre in Gdańsk)	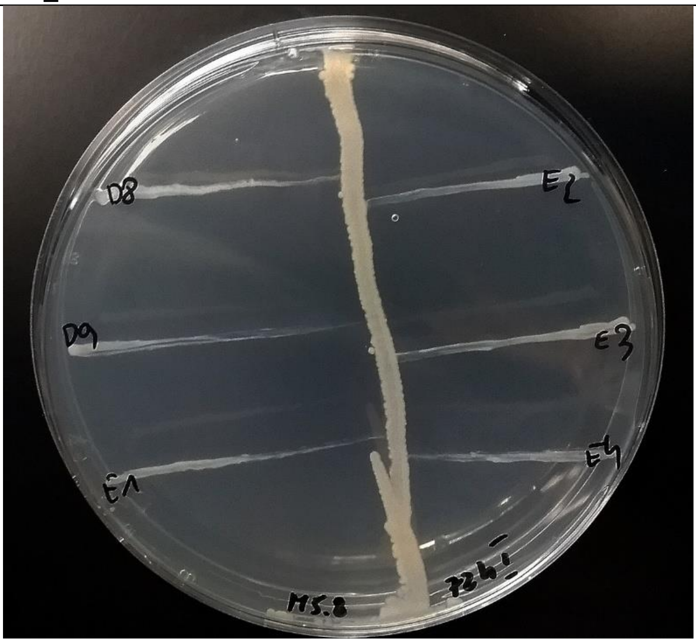<br>M5_8 72h I	*Candida guilliermondii* E2 (University Clinical Centre in Gdańsk)<br><br>*Candida guilliermondii* E3 (University Clinical Centre in Gdańsk)<br><br>*Candida albicans* E4 (University Clinical Centre in Gdańsk)

Candida albicans E5 (University Clinical Centre in Gdańsk)<br><br>*Candida glabrata* E6 (University Clinical Centre in Gdańsk)<br><br>*Candida glabrata* E7 (University Clinical Centre in Gdańsk)	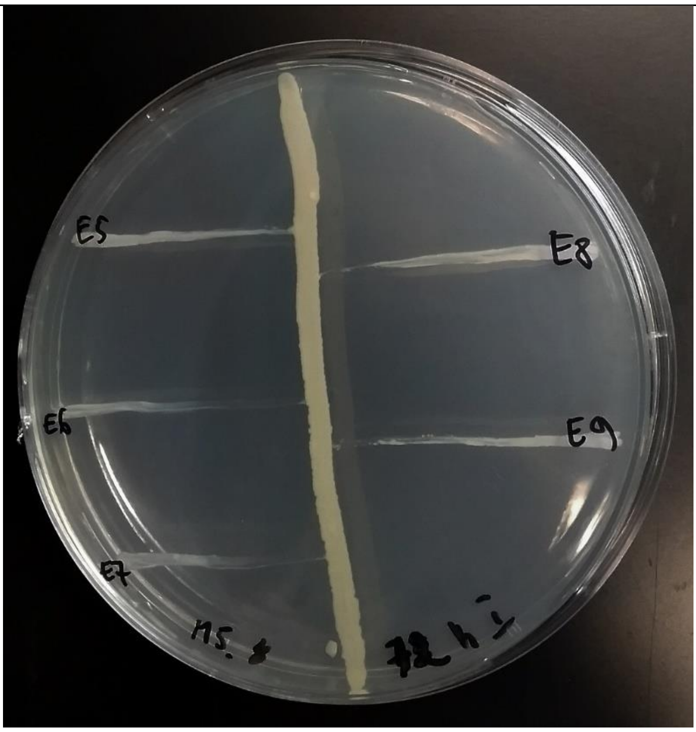<br>M5_8 72h I	*Candida* sp. E8 (University Clinical Centre in Gdańsk)<br><br>*Candida* sp. E9 (University Clinical Centre in Gdańsk)
Candida parapsilosis D2 (Bruss Laboratories, Gdynia, Poland)<br><br>*Candida glabrata* D3 (Bruss Laboratories, Gdynia, Poland)<br><br>*Candida tropicalis* D4 (Bruss Laboratories, Gdynia, Poland)	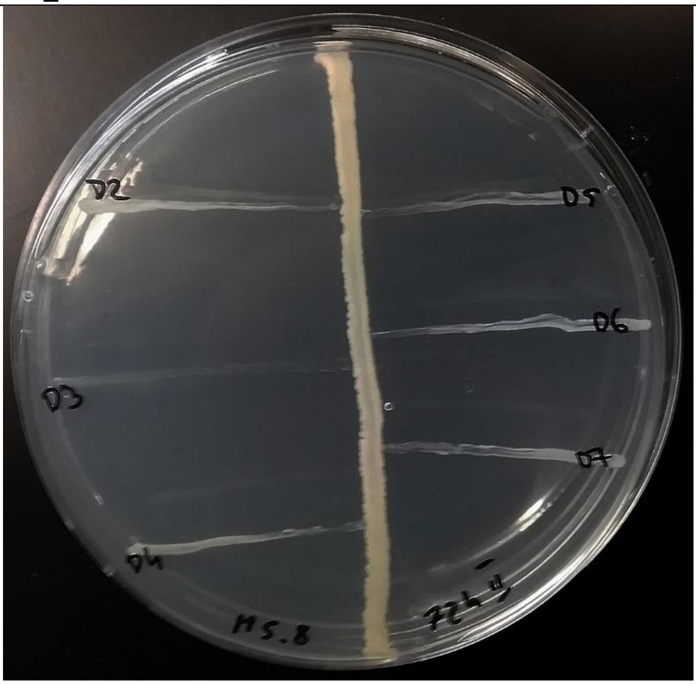<br>M5_8 72h II	*Candida dubliniensis* D5 (Bruss Laboratories, Gdynia, Poland)<br><br>*Candida albicans* D6 (Bruss Laboratories, Gdynia, Poland)<br><br>*Candida albicans* D7 (Medical University of Gdańsk)

Candida albicans D8 (Medical University of Gdańsk)<br><br>*Candida albicans* D9 (University Clinical Centre in Gdańsk)<br><br>*Candida albicans* E1 (University Clinical Centre in Gdańsk)	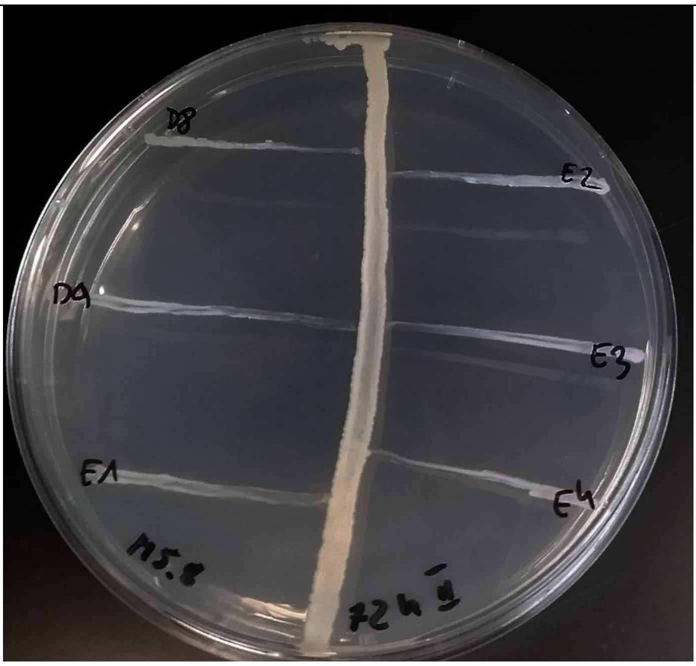<br>M5_8 72h II	*Candida guilliermondii* E2 (University Clinical Centre in Gdańsk)<br><br>*Candida guilliermondii* E3 (University Clinical Centre in Gdańsk)<br><br>*Candida albicans* E4 (University Clinical Centre in Gdańsk)
Candida albicans E5 (University Clinical Centre in Gdańsk)<br><br>*Candida glabrata* E6 (University Clinical Centre in Gdańsk)<br><br>*Candida glabrata* E7 (University Clinical Centre in Gdańsk)	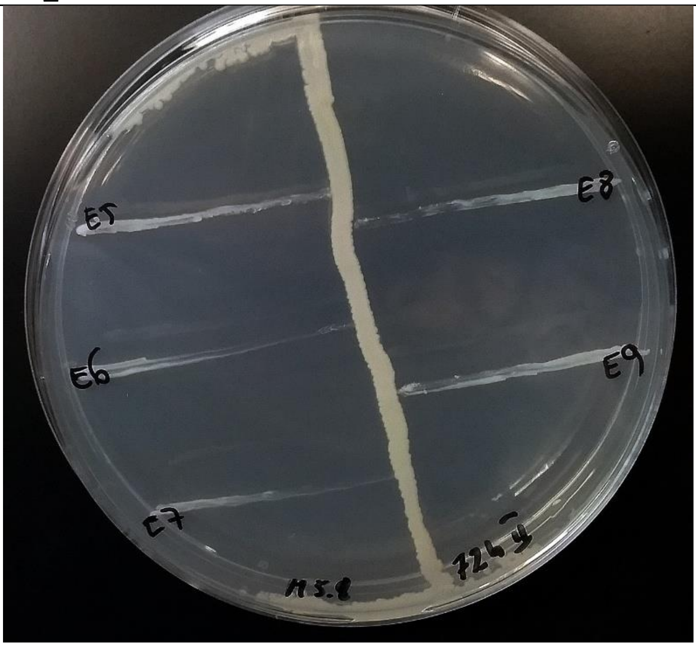<br>M5_8 72h II	*Candida* sp. E8 (University Clinical Centre in Gdańsk)<br><br>*Candida* sp. E9 (University Clinical Centre in Gdańsk)
Candida parapsilosis D2 (Bruss Laboratories, Gdynia, Poland)<br><br>*Candida glabrata* D3 (Bruss Laboratories, Gdynia, Poland)<br><br>*Candida tropicalis* D4 (Bruss Laboratories, Gdynia, Poland)	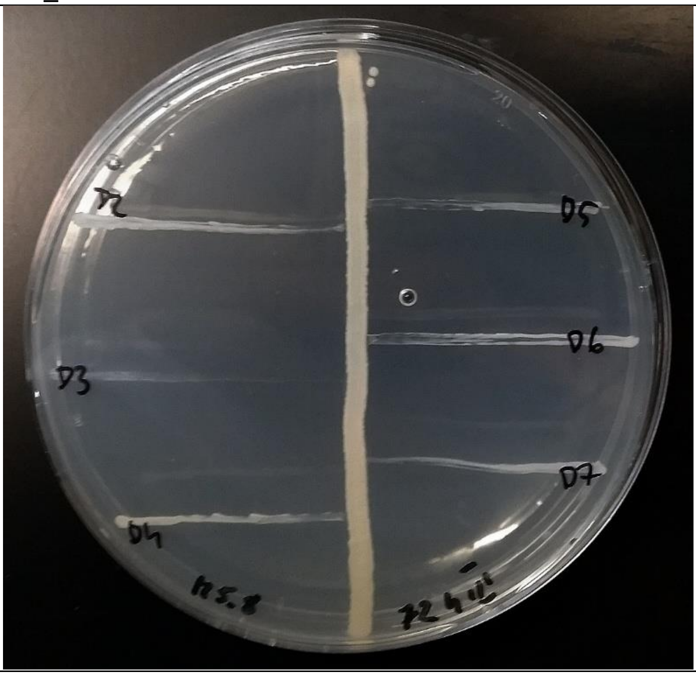	*Candida dubliniensis* D5 (Bruss Laboratories, Gdynia, Poland)<br><br>*Candida albicans* D6 (Bruss Laboratories, Gdynia, Poland)<br><br>*Candida albicans* D7 (Medical University of Gdańsk)

	M5_8 7h III	
Candida albicans D8 (Medical University of Gdańsk)<br><br>*Candida albicans* D9 (University Clinical Centre in Gdańsk)<br><br>*Candida albicans* E1 (University Clinical Centre in Gdańsk)	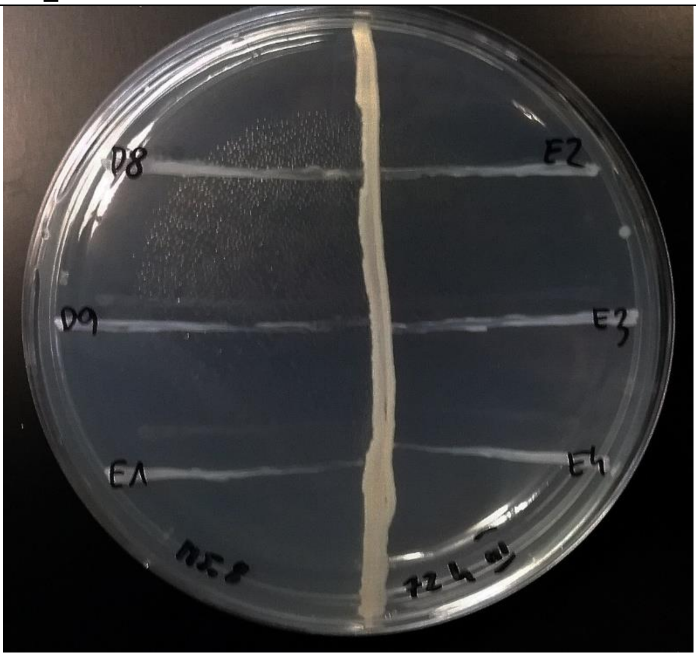<br>M5.8 72h III	*Candida guilliermondii* E2 (University Clinical Centre in Gdańsk)<br><br>*Candida guilliermondii* E3 (University Clinical Centre in Gdańsk)<br><br>*Candida albicans* E4 (University Clinical Centre in Gdańsk)
Candida albicans E5 (University Clinical Centre in Gdańsk)<br><br>*Candida glabrata* E6 (University Clinical Centre in Gdańsk)<br><br>*Candida glabrata* E7 (University Clinical Centre in Gdańsk)	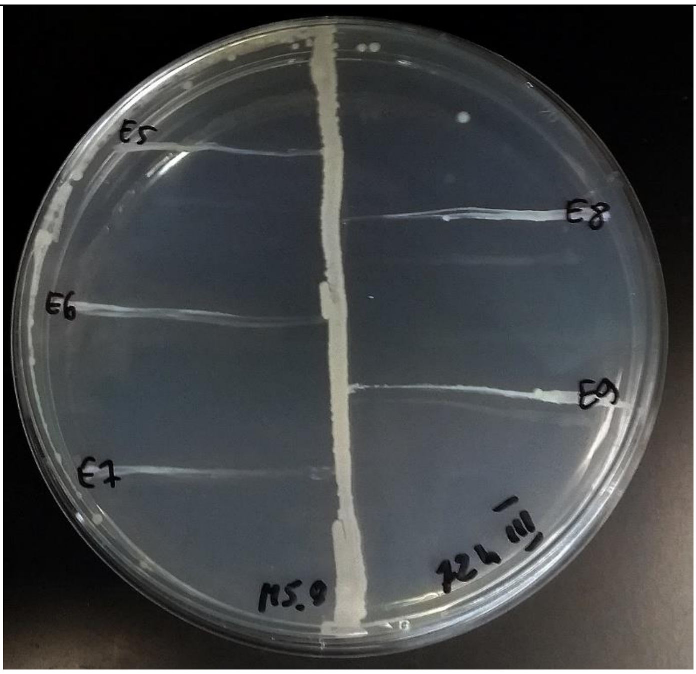<br>M5_8 72h III	*Candida* sp. E8 (University Clinical Centre in Gdańsk)<br><br>*Candida* sp. E9 (University Clinical Centre in Gdańsk)

Candida parapsilosis D2 (Bruss Laboratories, Gdynia, Poland)<br><br>*Candida glabrata* D3 (Bruss Laboratories, Gdynia, Poland)<br><br>*Candida tropicalis* D4 (Bruss Laboratories, Gdynia, Poland)	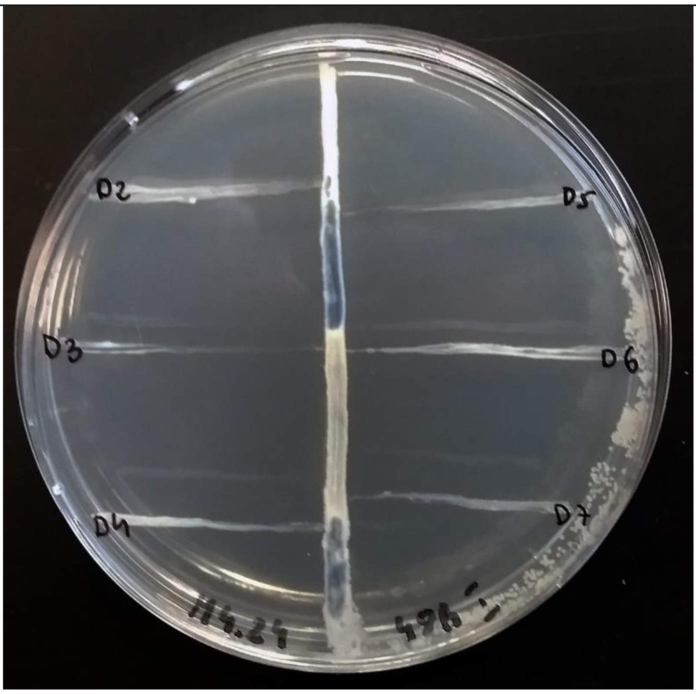<br>M4_24 48h I	*Candida dubliniensis* D5 (Bruss Laboratories, Gdynia, Poland)<br><br>*Candida albicans* D6 (Bruss Laboratories, Gdynia, Poland)<br><br>*Candida albicans* D7 (Medical University of Gdańsk)
Candida albicans D8 (Medical University of Gdańsk)<br><br>*Candida albicans* D9 (University Clinical Centre in Gdańsk)<br><br>*Candida albicans* E1 (University Clinical Centre in Gdańsk)	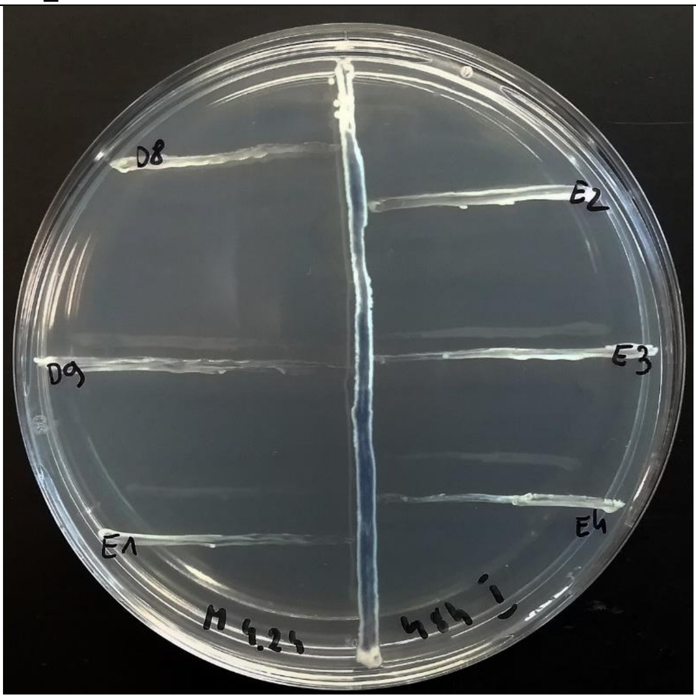<br>M4_24 48h I	*Candida guilliermondii* E2 (University Clinical Centre in Gdańsk)<br><br>*Candida guilliermondii* E3 (University Clinical Centre in Gdańsk)<br><br>*Candida albicans* E4 (University Clinical Centre in Gdańsk)

Candida albicans E5 (University Clinical Centre in Gdańsk)<br><br>*Candida glabrata* E6 (University Clinical Centre in Gdańsk)<br><br>*Candida glabrata* E7 (University Clinical Centre in Gdańsk)	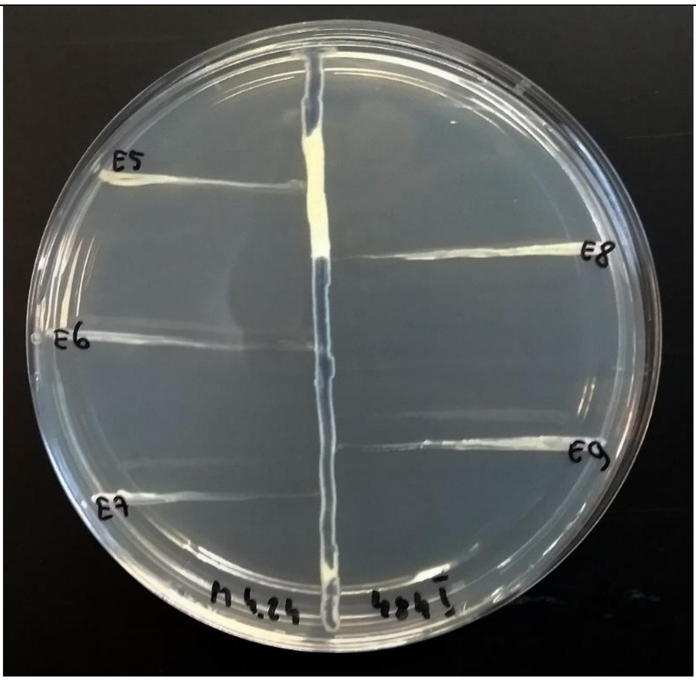<br>M4_24 48h I	*Candida* sp. E8 (University Clinical Centre in Gdańsk)<br><br>*Candida* sp. E9 (University Clinical Centre in Gdańsk)
Candida parapsilosis D2 (Bruss Laboratories, Gdynia, Poland)<br><br>*Candida glabrata* D3 (Bruss Laboratories, Gdynia, Poland)<br><br>*Candida tropicalis* D4 (Bruss Laboratories, Gdynia, Poland)	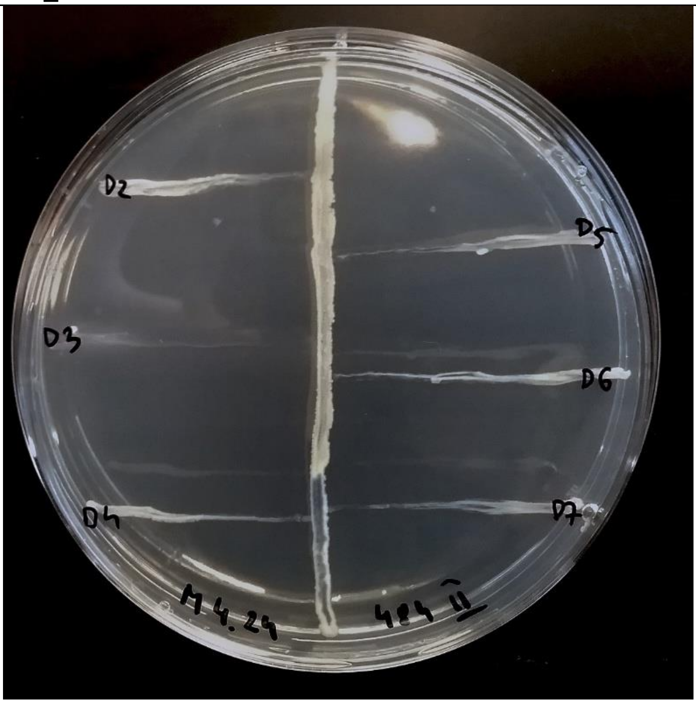<br>M4_24 48h II	*Candida dubliniensis* D5 (Bruss Laboratories, Gdynia, Poland)<br><br>*Candida albicans* D6 (Bruss Laboratories, Gdynia, Poland)<br><br>*Candida albicans* D7 (Medical University of Gdańsk)

Candida albicans D8 (Medical University of Gdańsk)<br><br>*Candida albicans* D9 (University Clinical Centre in Gdańsk)<br><br>*Candida albicans* E1 (University Clinical Centre in Gdańsk)	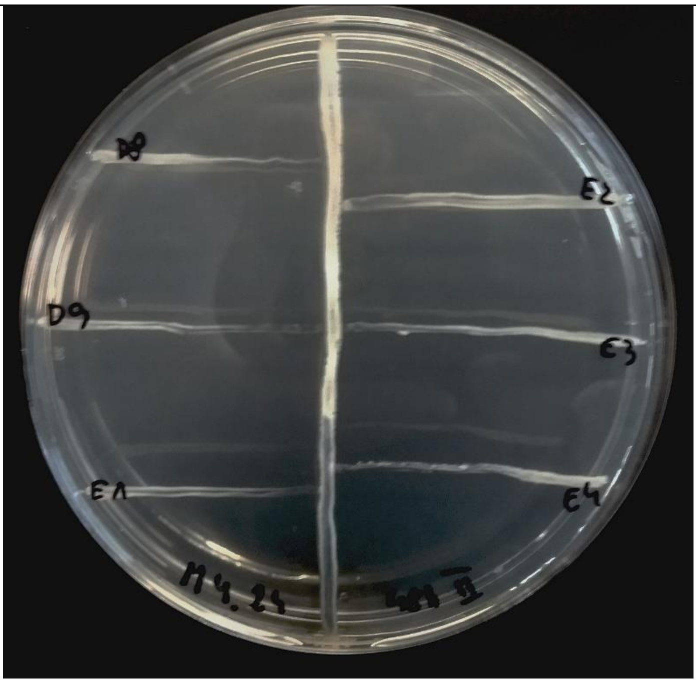<br><br>M4_24 48h II	*Candida guilliermondii* E2 (University Clinical Centre in Gdańsk)<br><br>*Candida guilliermondii* E3 (University Clinical Centre in Gdańsk)<br><br>*Candida albicans* E4 (University Clinical Centre in Gdańsk)
Candida albicans E5 (University Clinical Centre in Gdańsk)<br><br>*Candida glabrata* E6 (University Clinical Centre in Gdańsk)<br><br>*Candida glabrata* E7 (University Clinical Centre in Gdańsk)	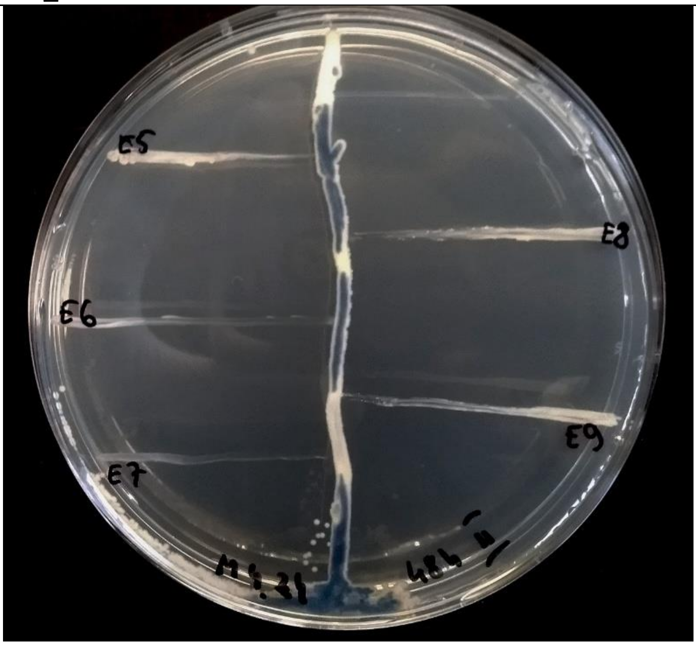<br><br>M4_24 48h II	*Candida* sp. E8 (University Clinical Centre in Gdańsk)<br><br>*Candida* sp. E9 (University Clinical Centre in Gdańsk)

Candida parapsilosis D2 (Bruss Laboratories, Gdynia, Poland)<br><br>*Candida glabrata* D3 (Bruss Laboratories, Gdynia, Poland)<br><br>*Candida tropicalis* D4 (Bruss Laboratories, Gdynia, Poland)	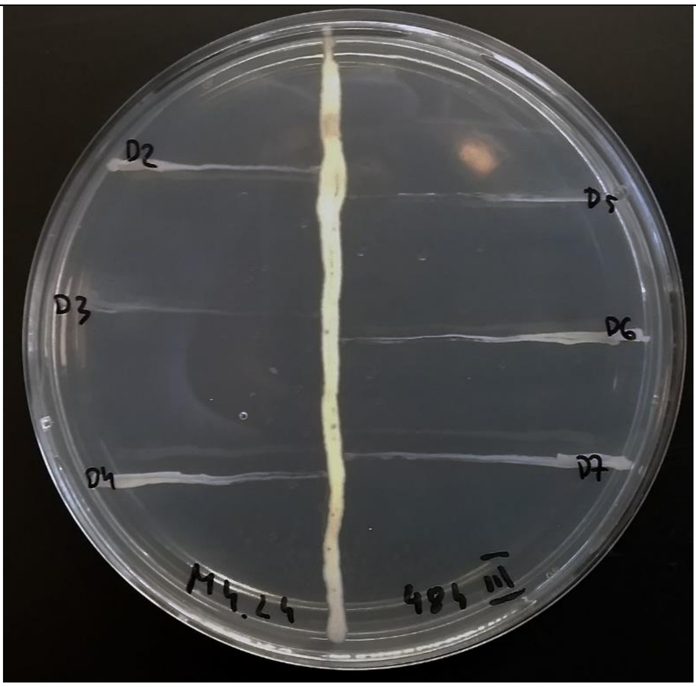<br>M4_24 48h III	*Candida dubliniensis* D5 (Bruss Laboratories, Gdynia, Poland)<br><br>*Candida albicans* D6 (Bruss Laboratories, Gdynia, Poland)<br><br>*Candida albicans* D7 (Medical University of Gdańsk)
Candida albicans D8 (Medical University of Gdańsk)<br><br>*Candida albicans* D9 (University Clinical Centre in Gdańsk)<br><br>*Candida albicans* E1 (University Clinical Centre in Gdańsk)	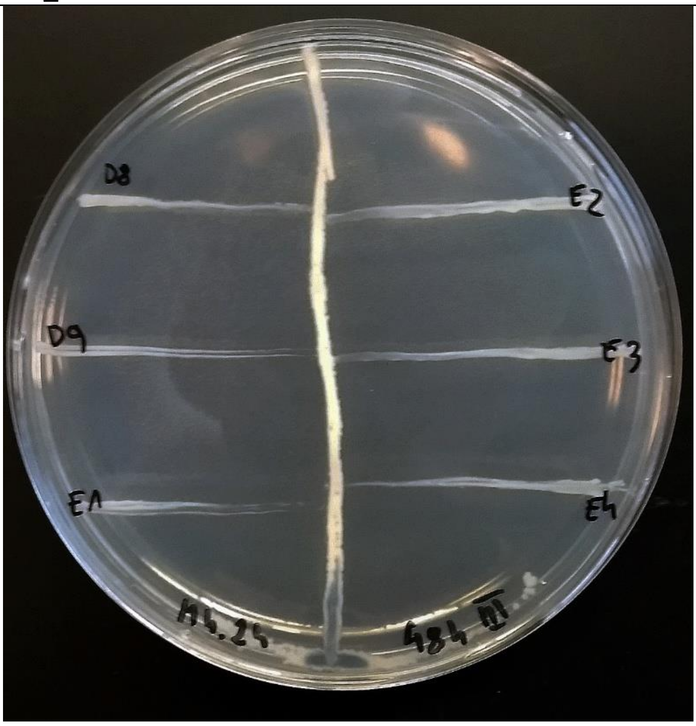<br>M4_24 48h III	*Candida guilliermondii* E2 (University Clinical Centre in Gdańsk)<br><br>*Candida guilliermondii* E3 (University Clinical Centre in Gdańsk)<br><br>*Candida albicans* E4 (University Clinical Centre in Gdańsk)

Candida albicans E5 (University Clinical Centre in Gdańsk)<br><br>*Candida glabrata* E6 (University Clinical Centre in Gdańsk)<br><br>*Candida glabrata* E7 (University Clinical Centre in Gdańsk)	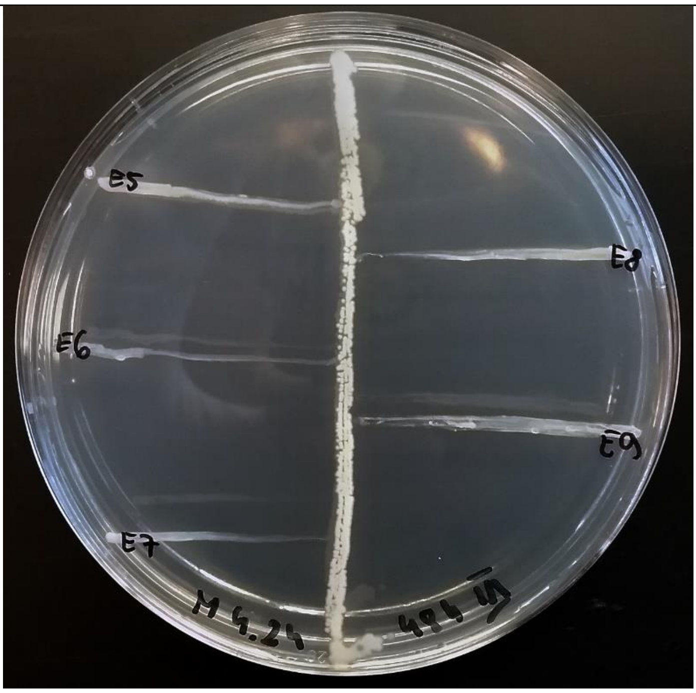<br>M4_24 48h III	*Candida* sp. E8 (University Clinical Centre in Gdańsk)<br><br>*Candida* sp. E9 (University Clinical Centre in Gdańsk)
Candida parapsilosis D2 (Bruss Laboratories, Gdynia, Poland)<br><br>*Candida glabrata* D3 (Bruss Laboratories, Gdynia, Poland)<br><br>*Candida tropicalis* D4 (Bruss Laboratories, Gdynia, Poland)	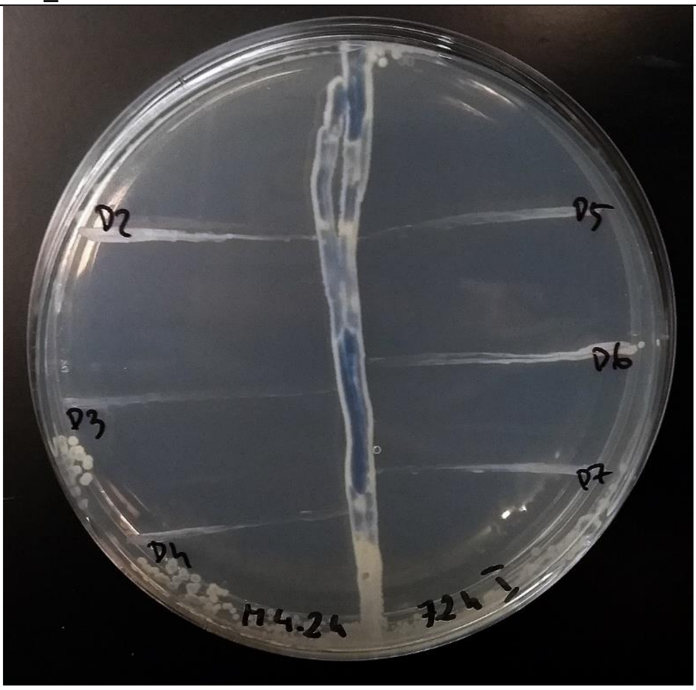<br>M4_24 72h I	*Candida dubliniensis* D5 (Bruss Laboratories, Gdynia, Poland)<br><br>*Candida albicans* D6 (Bruss Laboratories, Gdynia, Poland)<br><br>*Candida albicans* D7 (Medical University of Gdańsk)

Candida albicans D8 (Medical University of Gdańsk)<br><br>*Candida albicans* D9 (University Clinical Centre in Gdańsk)<br><br>*Candida albicans* E1 (University Clinical Centre in Gdańsk)	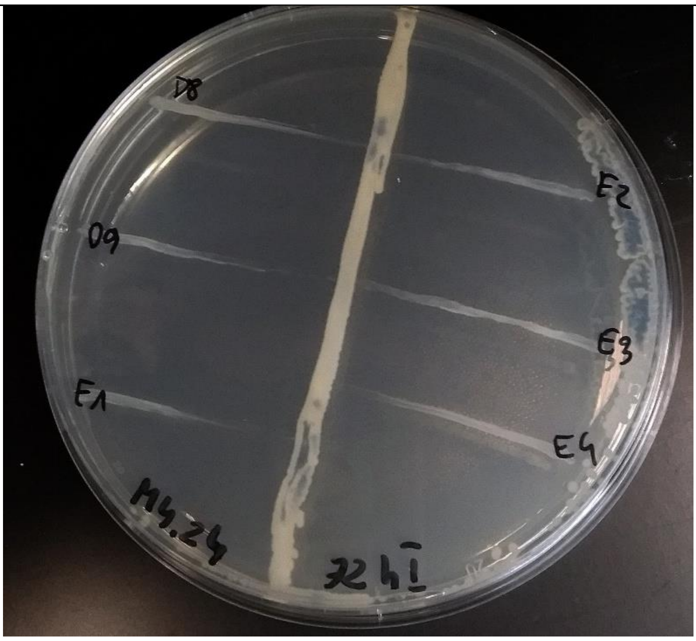<br>M4_24 72h I	*Candida guilliermondii* E2 (University Clinical Centre in Gdańsk)<br><br>*Candida guilliermondii* E3 (University Clinical Centre in Gdańsk)<br><br>*Candida albicans* E4 (University Clinical Centre in Gdańsk)
Candida albicans E5 (University Clinical Centre in Gdańsk)<br><br>*Candida glabrata* E6 (University Clinical Centre in Gdańsk)<br><br>*Candida glabrata* E7 (University Clinical Centre in Gdańsk)	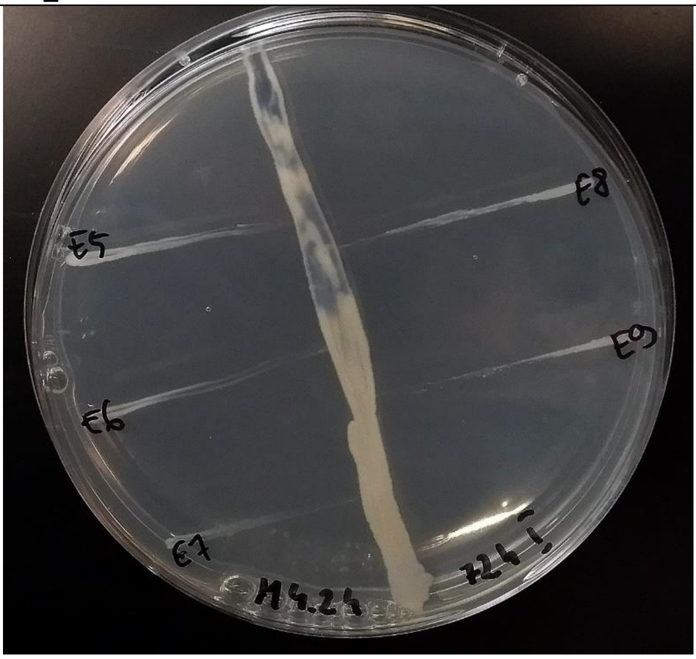<br>M4_24 72h I	*Candida* sp. E8 (University Clinical Centre in Gdańsk)<br><br>*Candida* sp. E9 (University Clinical Centre in Gdańsk)
Candida parapsilosis D2 (Bruss Laboratories, Gdynia, Poland)<br><br>*Candida glabrata* D3 (Bruss Laboratories, Gdynia, Poland)<br><br>*Candida tropicalis* D4 (Bruss Laboratories, Gdynia, Poland)	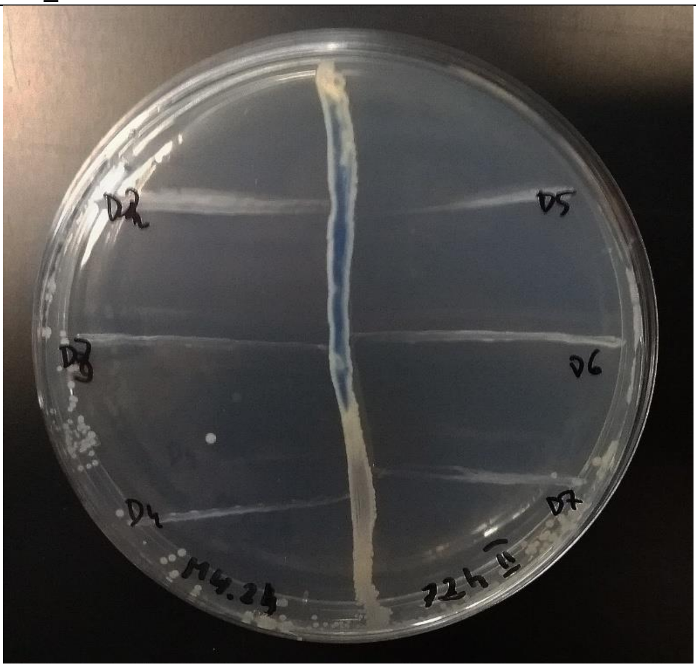<br>M4_24 72h II	*Candida dubliniensis* D5 (Bruss Laboratories, Gdynia, Poland)<br><br>*Candida albicans* D6 (Bruss Laboratories, Gdynia, Poland)<br><br>*Candida albicans* D7 (Medical University of Gdańsk)

Candida albicans D8 (Medical University of Gdańsk)<br><br>*Candida albicans* D9 (University Clinical Centre in Gdańsk)<br><br>*Candida albicans* E1 (University Clinical Centre in Gdańsk)	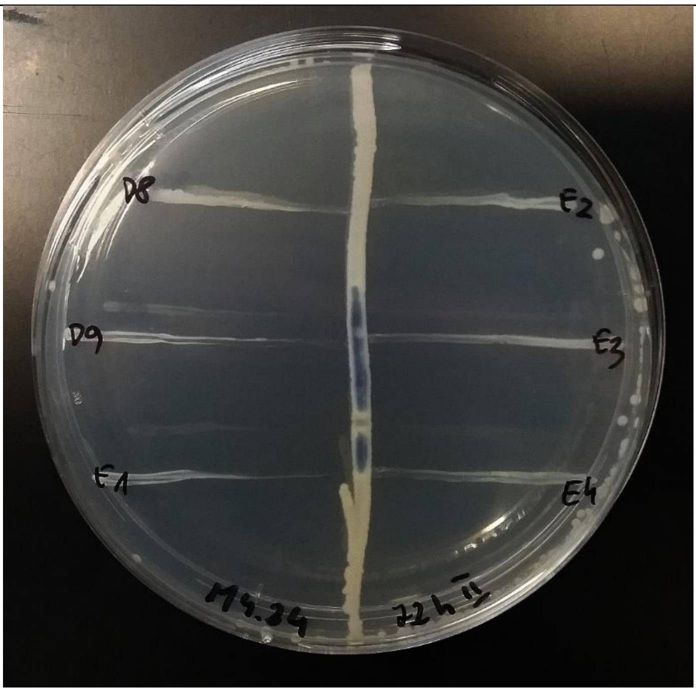<br>M4_24 72h II	*Candida guilliermondii* E2 (University Clinical Centre in Gdańsk)<br><br>*Candida guilliermondii* E3 (University Clinical Centre in Gdańsk)<br><br>*Candida albicans* E4 (University Clinical Centre in Gdańsk)
Candida albicans E5 (University Clinical Centre in Gdańsk)<br><br>*Candida glabrata* E6 (University Clinical Centre in Gdańsk)<br><br>*Candida glabrata* E7 (University Clinical Centre in Gdańsk)	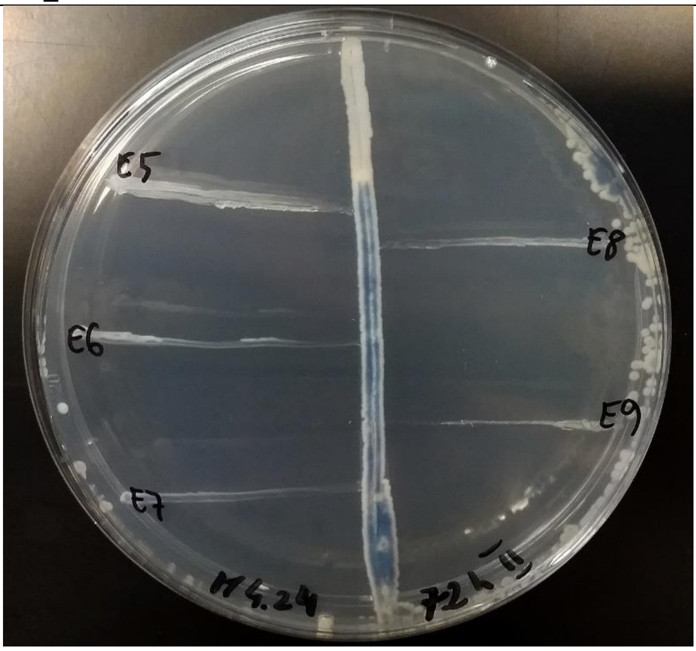<br>M4_24 72h II	*Candida* sp. E8 (University Clinical Centre in Gdańsk)<br><br>*Candida* sp. E9 (University Clinical Centre in Gdańsk)

Candida parapsilosis D2 (Bruss Laboratories, Gdynia, Poland)<br><br>*Candida glabrata* D3 (Bruss Laboratories, Gdynia, Poland)<br><br>*Candida tropicalis* D4 (Bruss Laboratories, Gdynia, Poland)	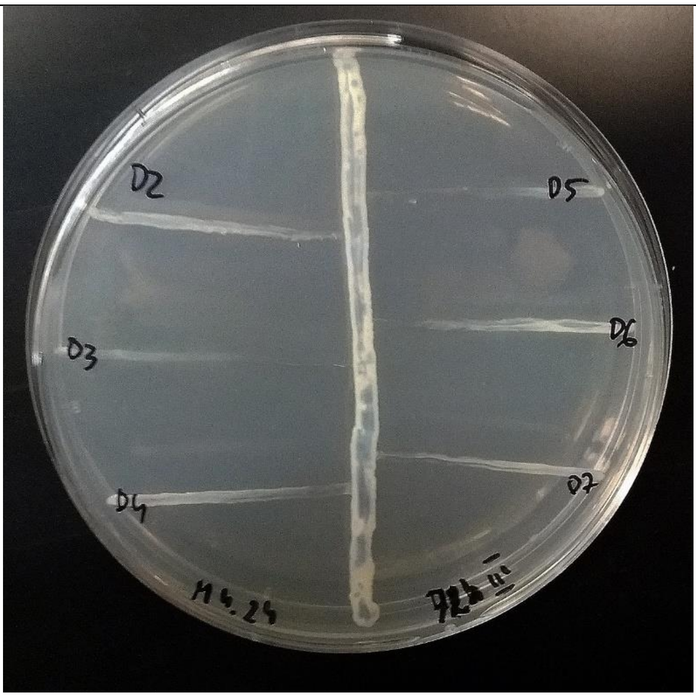<br>M4_24 72h III	*Candida dubliniensis* D5 (Bruss Laboratories, Gdynia, Poland)<br><br>*Candida albicans* D6 (Bruss Laboratories, Gdynia, Poland)<br><br>*Candida albicans* D7 (Medical University of Gdańsk)
Candida albicans D8 (Medical University of Gdańsk)<br><br>*Candida albicans* D9 (University Clinical Centre in Gdańsk)<br><br>*Candida albicans* E1 (University Clinical Centre in Gdańsk)	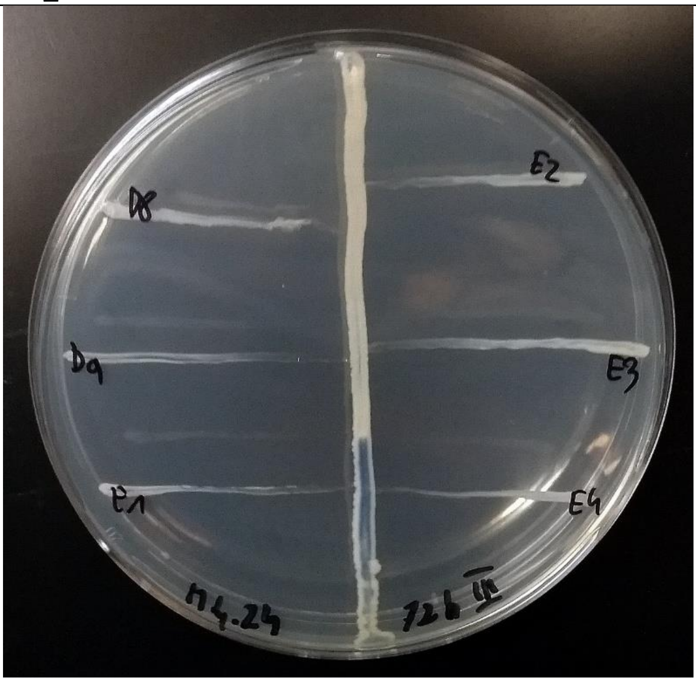<br>M4_24 72h III	*Candida guilliermondii* E2 (University Clinical Centre in Gdańsk)<br><br>*Candida guilliermondii* E3 (University Clinical Centre in Gdańsk)<br><br>*Candida albicans* E4 (University Clinical Centre in Gdańsk)

Candida albicans E5 (University Clinical Centre in Gdańsk)<br><br>*Candida glabrata* E6 (University Clinical Centre in Gdańsk)<br><br>*Candida glabrata* E7 (University Clinical Centre in Gdańsk)	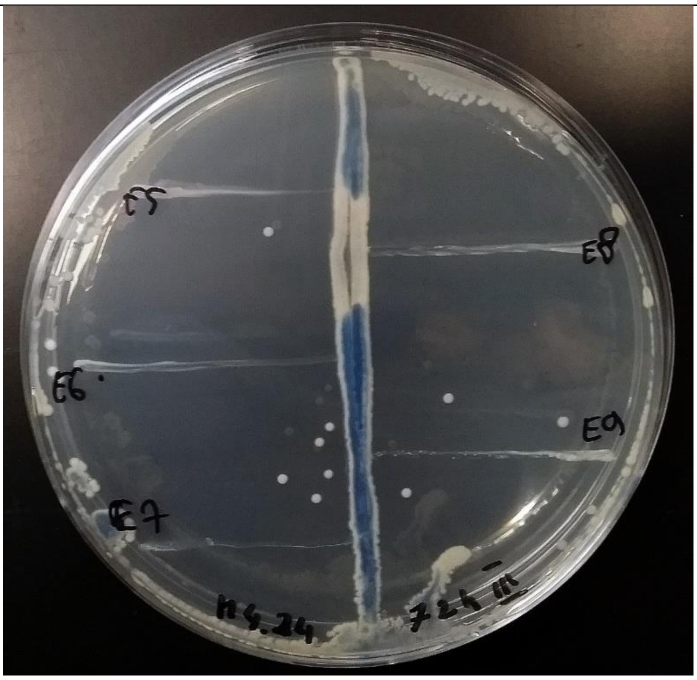<br>M4_24 72h III	*Candida* sp. E8 (University Clinical Centre in Gdańsk)<br><br>*Candida* sp. E9 (University Clinical Centre in Gdańsk)
Staphylococcus aureus MRSA 297 (Medical University of Gdańsk)<br><br>*Staphylococcus aureus* MRSA 202 (Medical University of Gdańsk)<br><br>*Staphylococcus aureus* MRSA 342 (Medical University of Gdańsk)<br><br>*Staphylococcus aureus* MRSA 199 (Medical University of Gdańsk)	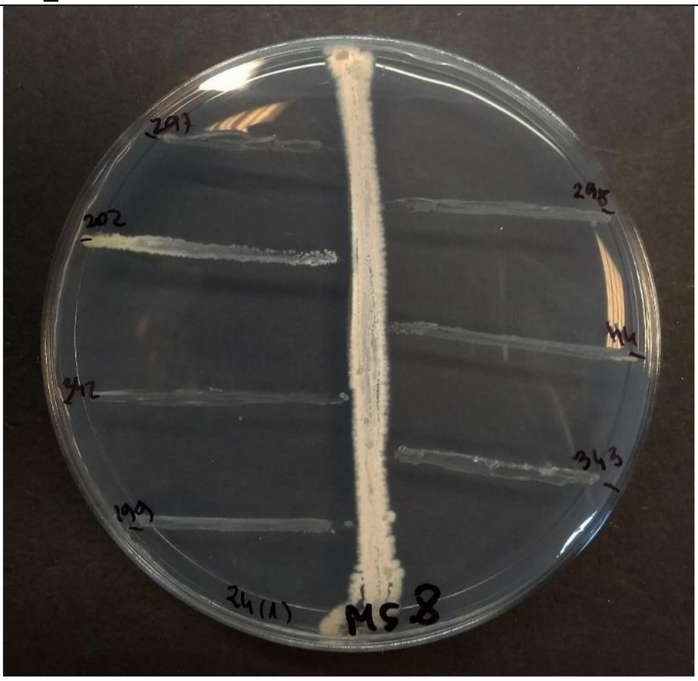<br>M5_8 24h I	*Staphylococcus aureus* MRSA 298 (Medical University of Gdańsk)<br><br>*Staphylococcus aureus* MRSA 44 (Medical University of Gdańsk)<br><br>*Staphylococcus aureus* MRSA 343 (Medical University of Gdańsk)

<table>
<tr>
<td>Staphylococcus aureus
MRSA 352 (Medical University of Gdańsk)

Staphylococcus aureus
MRSA 116 (Medical University of Gdańsk)

Staphylococcus aureus
MRSA 115 (Medical University of Gdańsk)

Staphylococcus aureus
MRSA 122 (Medical University of Gdańsk)</td>
<td>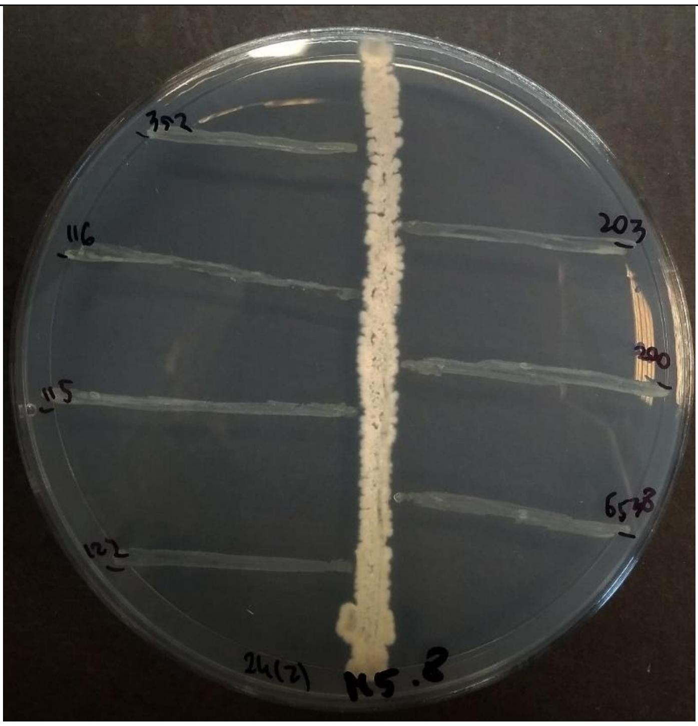

M5_8 24h II</td>
<td>Staphylococcus aureus
MRSA 203 (Medical University of Gdańsk)

Staphylococcus aureus
MRSA 200 (Medical University of Gdańsk)

Staphylococcus aureus
MRSA ATCC 6538 (Medical University of Gdańsk)</td>
</tr>
<tr>
<td>Staphylococcus aureus
MRSA 271 (Medical University of Gdańsk)

Staphylococcus aureus
MRSA 108 (Medical University of Gdańsk)

Staphylococcus aureus
MRSA 124 (Medical University of Gdańsk)

Staphylococcus aureus
MRSA 149 (Medical University of Gdańsk)</td>
<td>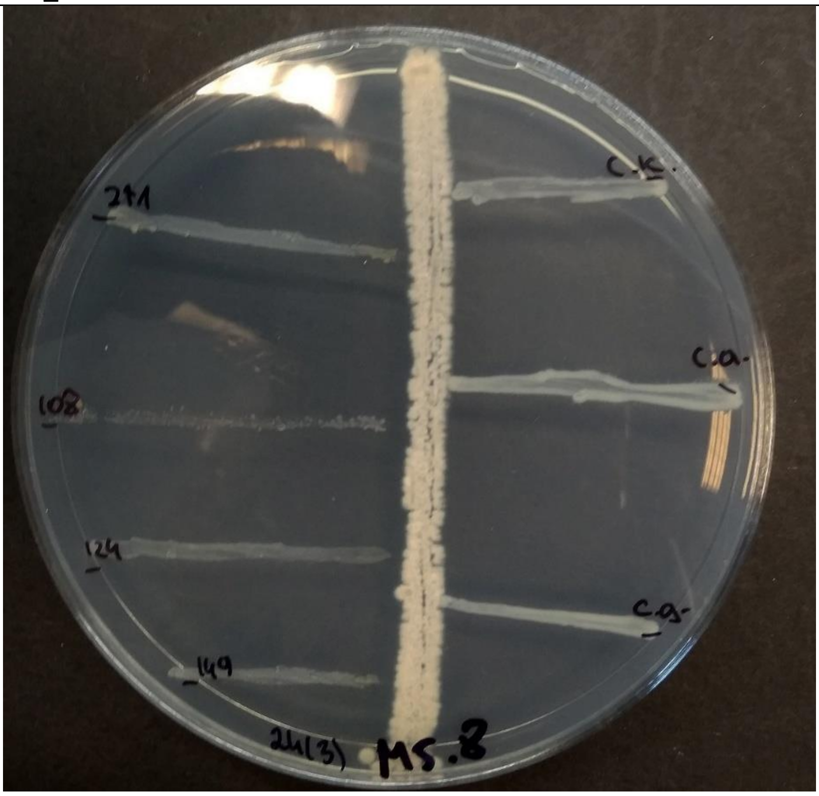

M5_8 24h III</td>
<td>-
-
-
(to są candida nieopisane w publikacji)</td>
</tr>
</table>

Staphylococcus aureus MRSA 297 (Medical University of Gdańsk)<br><br>*Staphylococcus aureus* MRSA 202 (Medical University of Gdańsk)<br><br>*Staphylococcus aureus* MRSA 342 (Medical University of Gdańsk)<br><br>*Staphylococcus aureus* MRSA 199 (Medical University of Gdańsk)	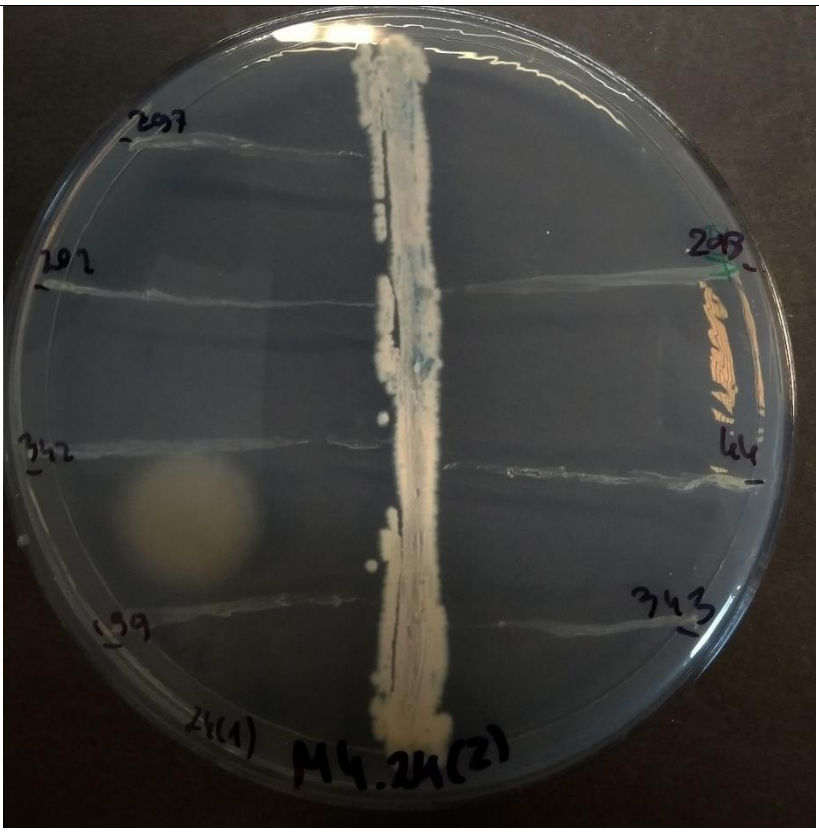<br>M4_24 24h I	*Staphylococcus aureus* MRSA 298 (Medical University of Gdańsk)<br><br>*Staphylococcus aureus* MRSA 44 (Medical University of Gdańsk)<br><br>*Staphylococcus aureus* MRSA 343 (Medical University of Gdańsk)
Staphylococcus aureus MRSA 352 (Medical University of Gdańsk)<br><br>*Staphylococcus aureus* MRSA 116 (Medical University of Gdańsk)<br><br>*Staphylococcus aureus* MRSA 115 (Medical University of Gdańsk)<br><br>*Staphylococcus aureus* MRSA 122 (Medical University of Gdańsk)	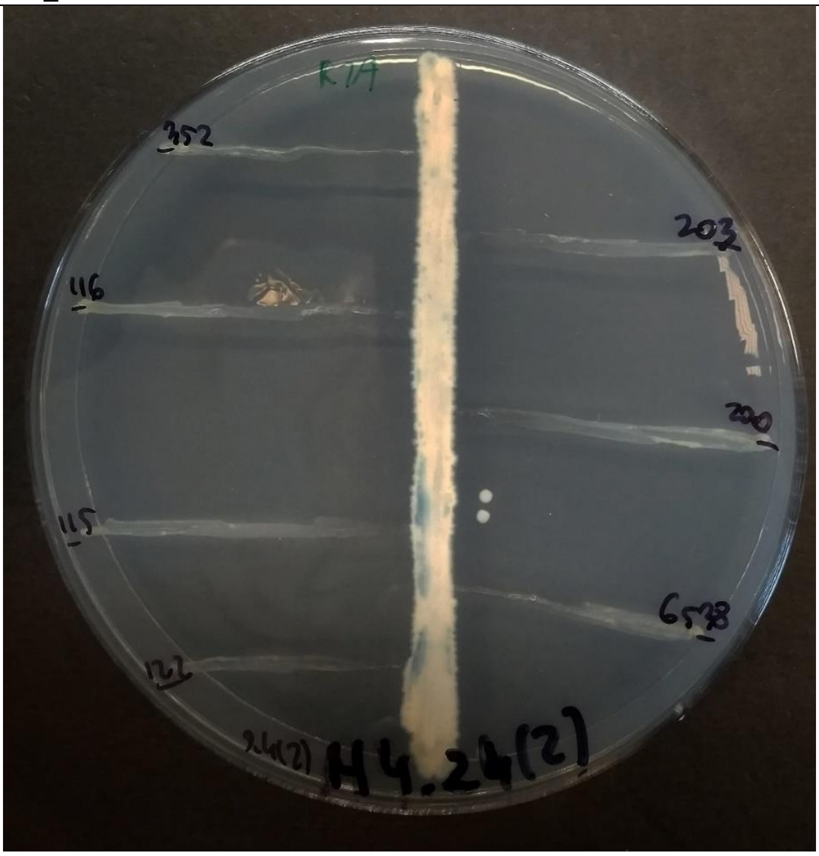<br>M4_24 24h II	*Staphylococcus aureus* MRSA 203 (Medical University of Gdańsk)<br><br>*Staphylococcus aureus* MRSA 200 (Medical University of Gdańsk)<br><br>*Staphylococcus aureus* MRSA ATCC 6538 (Medical University of Gdańsk)

<table>
<tr>
<td>Staphylococcus aureus
MRSA 271 (Medical University of Gdańsk)

Staphylococcus aureus
MRSA 108 (Medical University of Gdańsk)

Staphylococcus aureus
MRSA 124 (Medical University of Gdańsk)

Staphylococcus aureus
MRSA 149 (Medical University of Gdańsk)</td>
<td>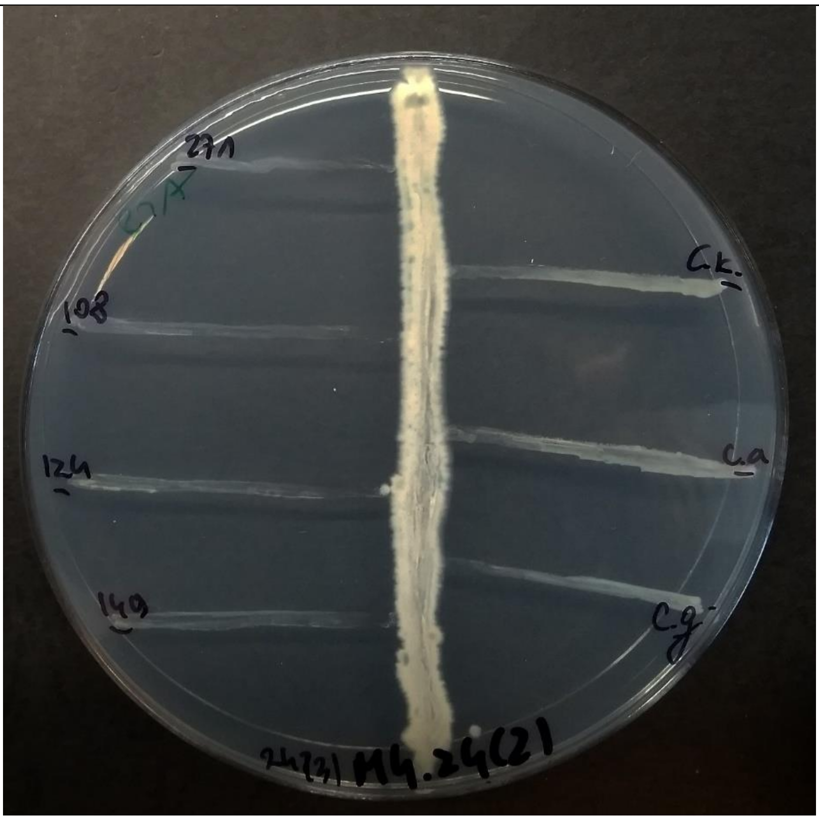

M4_24 24h III</td>
<td>-
-
-
(to są candida nieopisane w publikacji)</td>
</tr>
<tr>
<td>Staphylococcus aureus
MRSA 297 (Medical University of Gdańsk)

Staphylococcus aureus
MRSA 202 (Medical University of Gdańsk)

Staphylococcus aureus
MRSA 342 (Medical University of Gdańsk)

Staphylococcus aureus
MRSA 199 (Medical University of Gdańsk)</td>
<td>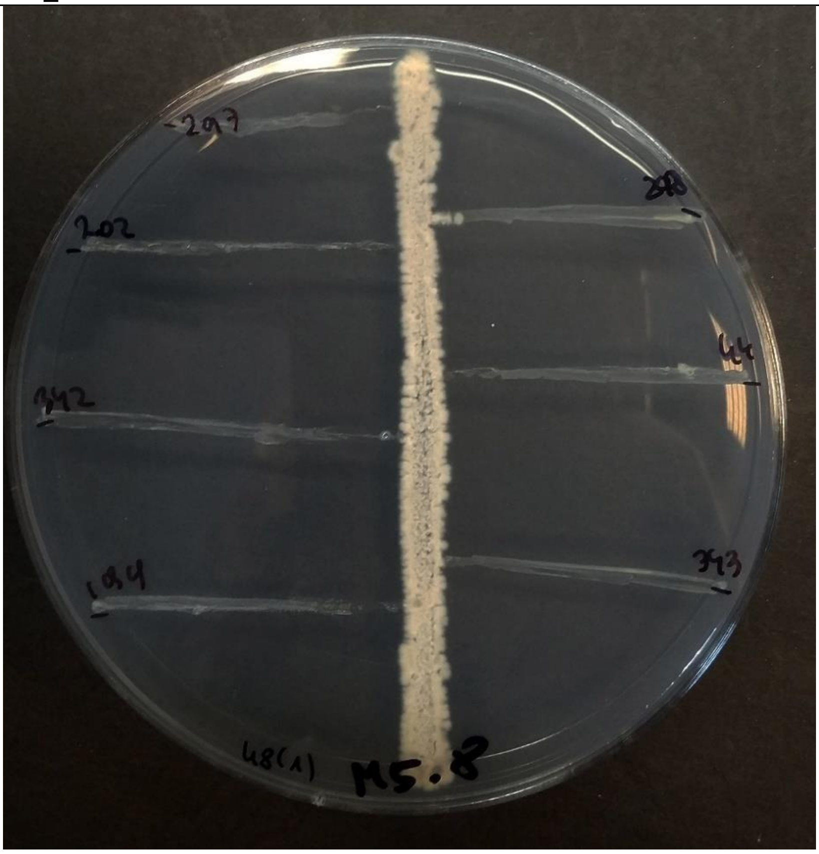

M5_8 48h I</td>
<td>Staphylococcus aureus
MRSA 298 (Medical University of Gdańsk)

Staphylococcus aureus
MRSA 44 (Medical University of Gdańsk)

Staphylococcus aureus
MRSA 343 (Medical University of Gdańsk)</td>
</tr>
</table>

Staphylococcus aureus MRSA 352 (Medical University of Gdańsk)<br><br>*Staphylococcus aureus* MRSA 116 (Medical University of Gdańsk)<br><br>*Staphylococcus aureus* MRSA 115 (Medical University of Gdańsk)<br><br>*Staphylococcus aureus* MRSA 122 (Medical University of Gdańsk)	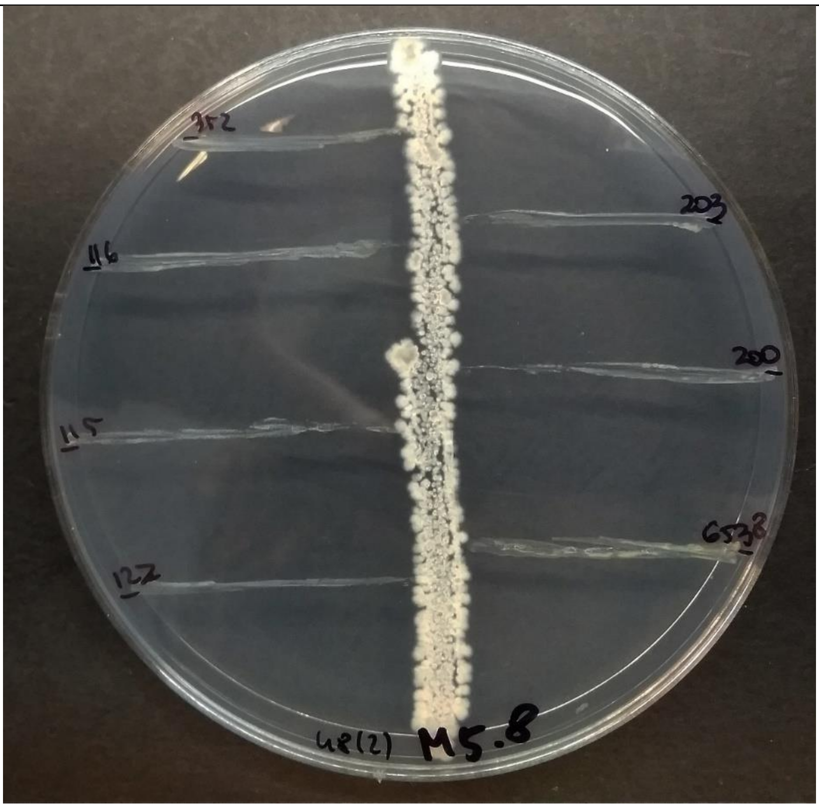<br>M5_8 48h II	*Staphylococcus aureus* MRSA 203 (Medical University of Gdańsk)<br><br>*Staphylococcus aureus* MRSA 200 (Medical University of Gdańsk)<br><br>*Staphylococcus aureus* MRSA ATCC 6538 (Medical University of Gdańsk)
Staphylococcus aureus MRSA 271 (Medical University of Gdańsk)<br><br>*Staphylococcus aureus* MRSA 108 (Medical University of Gdańsk)<br><br>*Staphylococcus aureus* MRSA 124 (Medical University of Gdańsk)<br><br>*Staphylococcus aureus* MRSA 149 (Medical University of Gdańsk)	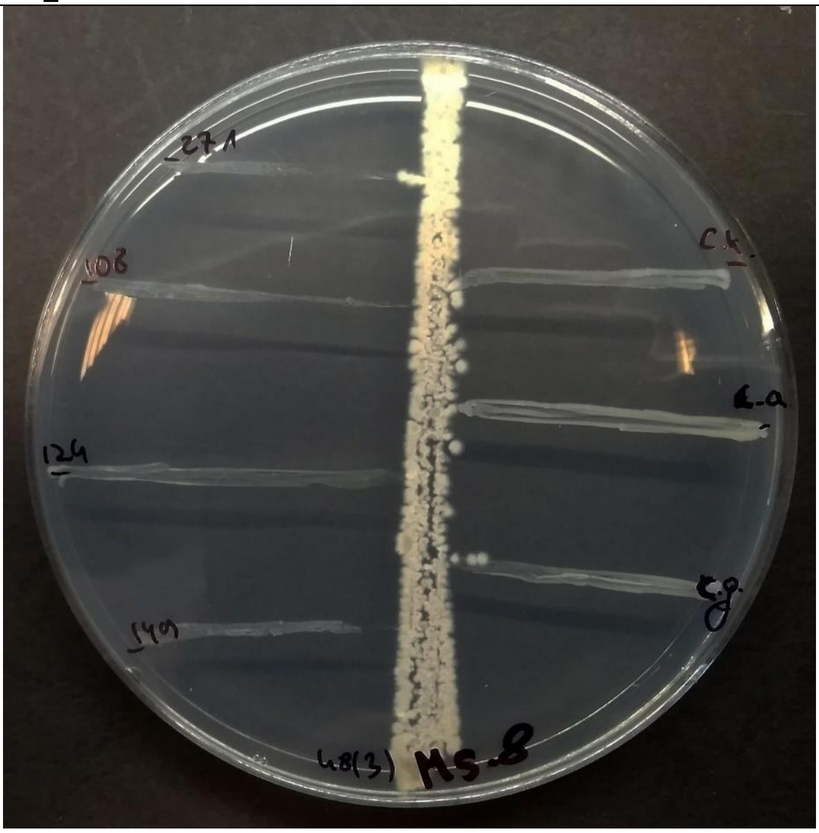<br>M5_8 48h III	-<br>-<br>-<br>(to są candida nieopisane w publikacji)

Staphylococcus aureus<br>MRSA 297 (Medical University of Gdańsk)<br><br>*Staphylococcus aureus*<br>MRSA 202 (Medical University of Gdańsk)<br><br>*Staphylococcus aureus*<br>MRSA 342 (Medical University of Gdańsk)<br><br>*Staphylococcus aureus*<br>MRSA 199 (Medical University of Gdańsk)	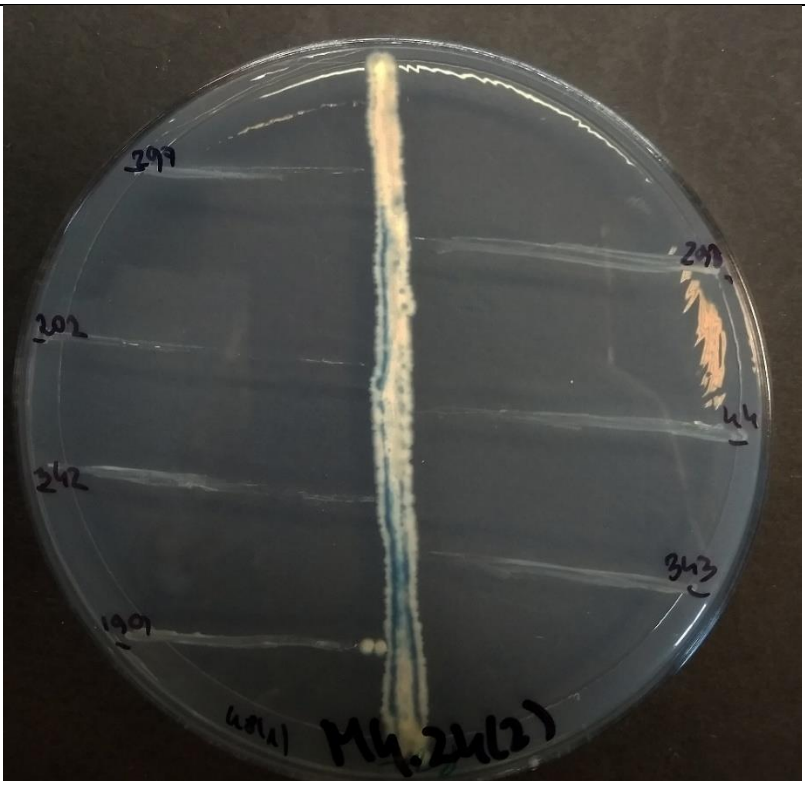<br>M4_24 48h I	*Staphylococcus aureus*<br>MRSA 298 (Medical University of Gdańsk)<br><br>*Staphylococcus aureus*<br>MRSA 44 (Medical University of Gdańsk)<br><br>*Staphylococcus aureus*<br>MRSA 343 (Medical University of Gdańsk)
Staphylococcus aureus<br>MRSA 352 (Medical University of Gdańsk)<br><br>*Staphylococcus aureus*<br>MRSA 116 (Medical University of Gdańsk)<br><br>*Staphylococcus aureus*<br>MRSA 115 (Medical University of Gdańsk)<br><br>*Staphylococcus aureus*<br>MRSA 122 (Medical University of Gdańsk)	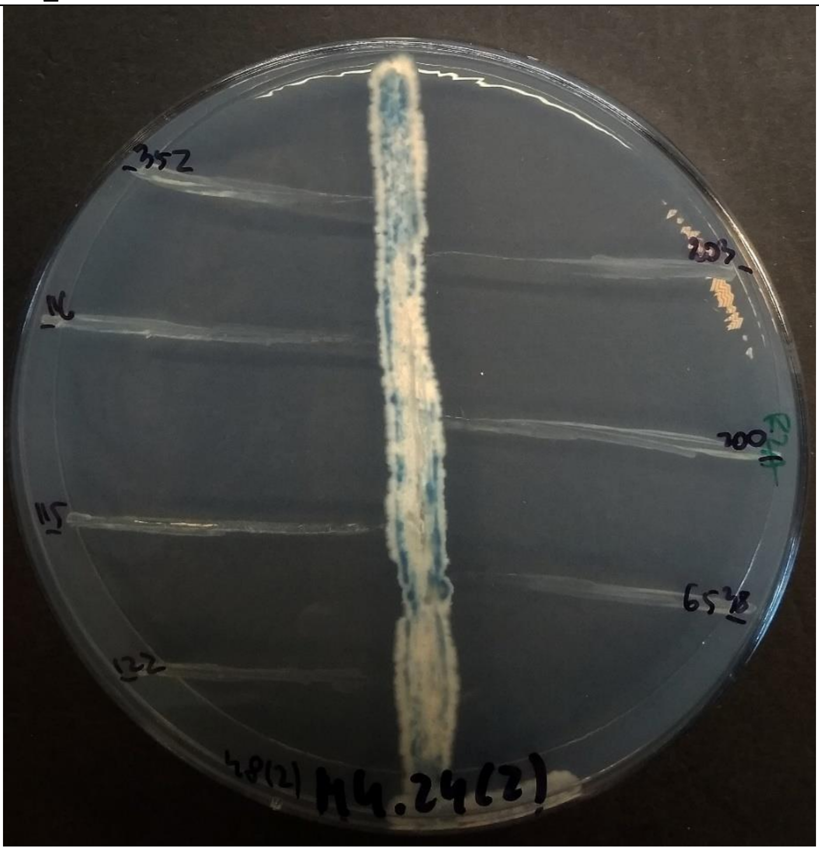<br>M4_24 48h II	*Staphylococcus aureus*<br>MRSA 203 (Medical University of Gdańsk)<br><br>*Staphylococcus aureus*<br>MRSA 200 (Medical University of Gdańsk)<br><br>*Staphylococcus aureus*<br>MRSA ATCC 6538 (Medical University of Gdańsk)

Staphylococcus aureus MRSA 271 (Medical University of Gdańsk)<br><br>*Staphylococcus aureus* MRSA 108 (Medical University of Gdańsk)<br><br>*Staphylococcus aureus* MRSA 124 (Medical University of Gdańsk)<br><br>*Staphylococcus aureus* MRSA 149 (Medical University of Gdańsk)	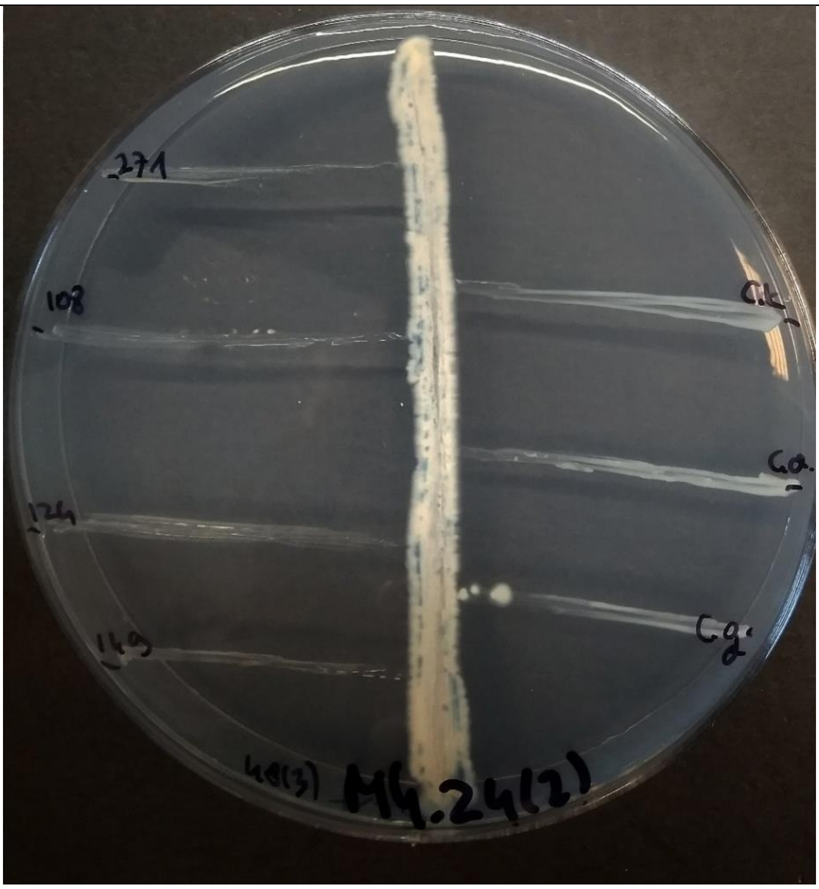<br>M4_24 48h III	-<br>-<br>-<br>(to są candida nieopisane w publikacji)
Staphylococcus aureus MRSA 297 (Medical University of Gdańsk)<br><br>*Staphylococcus aureus* MRSA 202 (Medical University of Gdańsk)<br><br><br>*Staphylococcus aureus* MRSA 342 (Medical University of Gdańsk)<br><br>*Staphylococcus aureus* MRSA 199 (Medical University of Gdańsk)	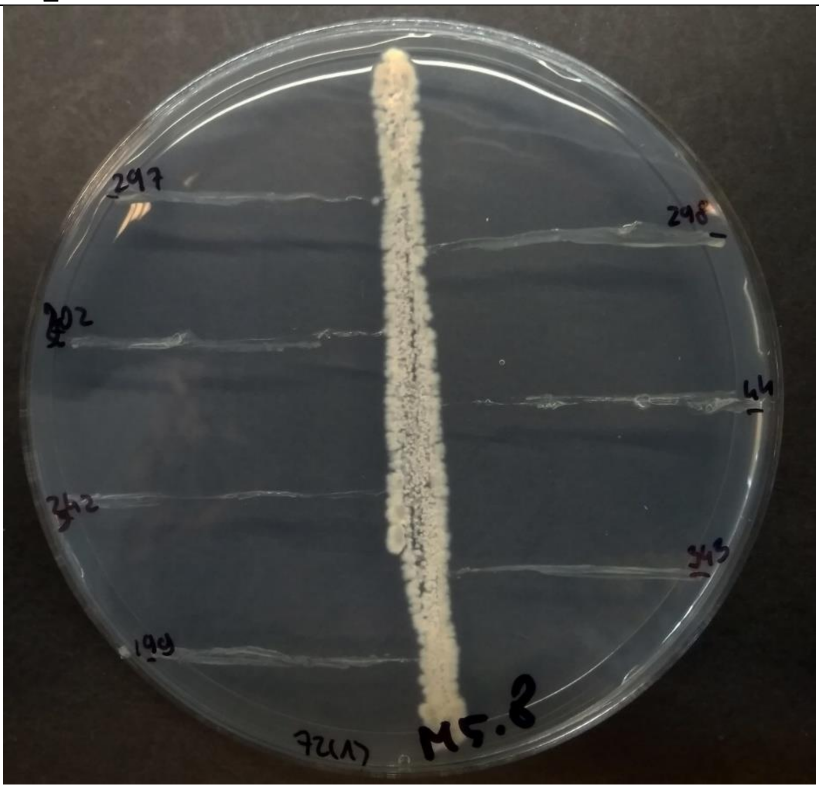<br>M5_8 72h I	*Staphylococcus aureus* MRSA 298 (Medical University of Gdańsk)<br><br>*Staphylococcus aureus* MRSA 44 (Medical University of Gdańsk)<br><br>*Staphylococcus aureus* MRSA 343 (Medical University of Gdańsk)

Staphylococcus aureus<br>MRSA 352 (Medical University of Gdańsk)<br><br>*Staphylococcus aureus*<br>MRSA 116 (Medical University of Gdańsk)<br><br>*Staphylococcus aureus*<br>MRSA 115 (Medical University of Gdańsk)<br><br>*Staphylococcus aureus*<br>MRSA 122 (Medical University of Gdańsk)	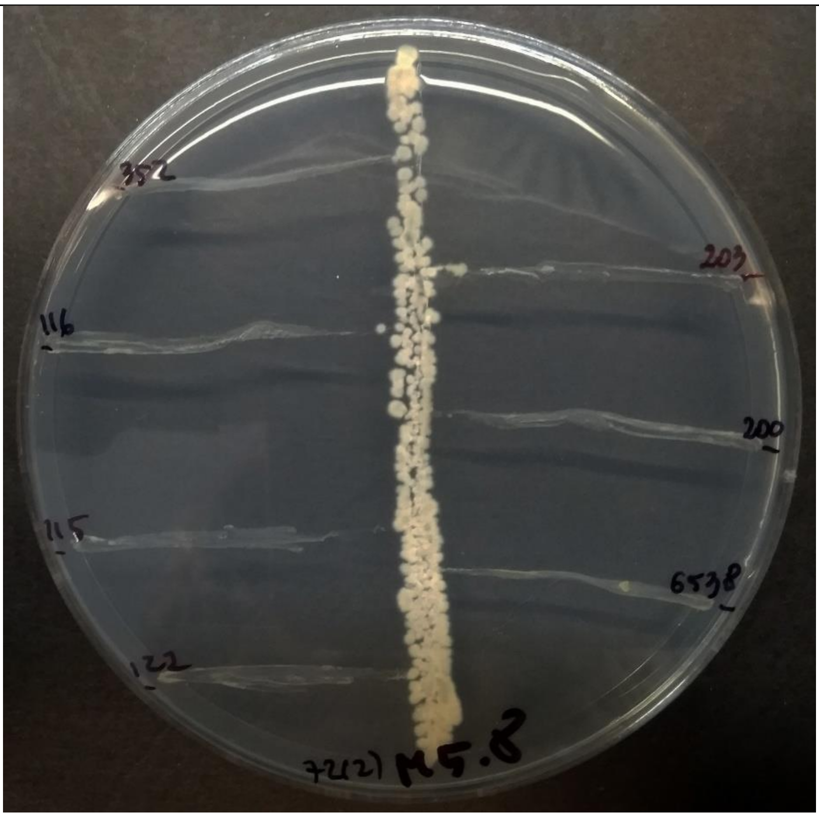<br>M5_8 72h II	*Staphylococcus aureus*<br>MRSA 203 (Medical University of Gdańsk)<br><br>*Staphylococcus aureus*<br>MRSA 200 (Medical University of Gdańsk)<br><br>*Staphylococcus aureus*<br>MRSA ATCC 6538 (Medical University of Gdańsk)
Staphylococcus aureus<br>MRSA 271 (Medical University of Gdańsk)<br><br>*Staphylococcus aureus*<br>MRSA 108 (Medical University of Gdańsk)<br><br>*Staphylococcus aureus*<br>MRSA 124 (Medical University of Gdańsk)<br><br>*Staphylococcus aureus*<br>MRSA 149 (Medical University of Gdańsk)	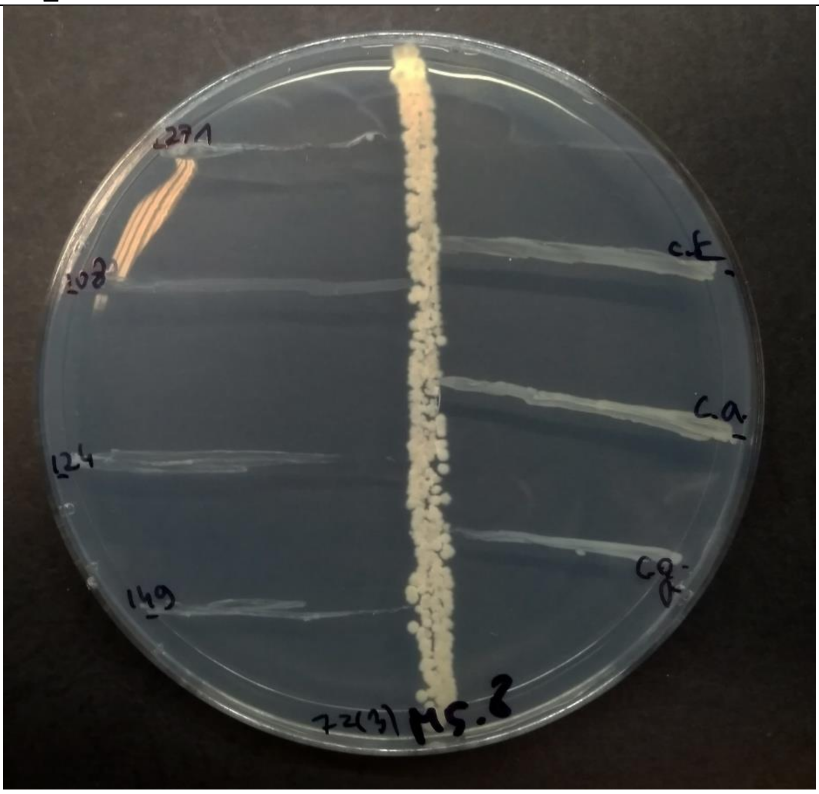<br>M5_8 72h III	-<br>-<br>-<br>(to są candida nieopisane w publikacji)

Staphylococcus aureus MRSA 297 (Medical University of Gdańsk)<br><br>*Staphylococcus aureus* MRSA 202 (Medical University of Gdańsk)<br><br>*Staphylococcus aureus* MRSA 342 (Medical University of Gdańsk)<br><br>*Staphylococcus aureus* MRSA 199 (Medical University of Gdańsk)	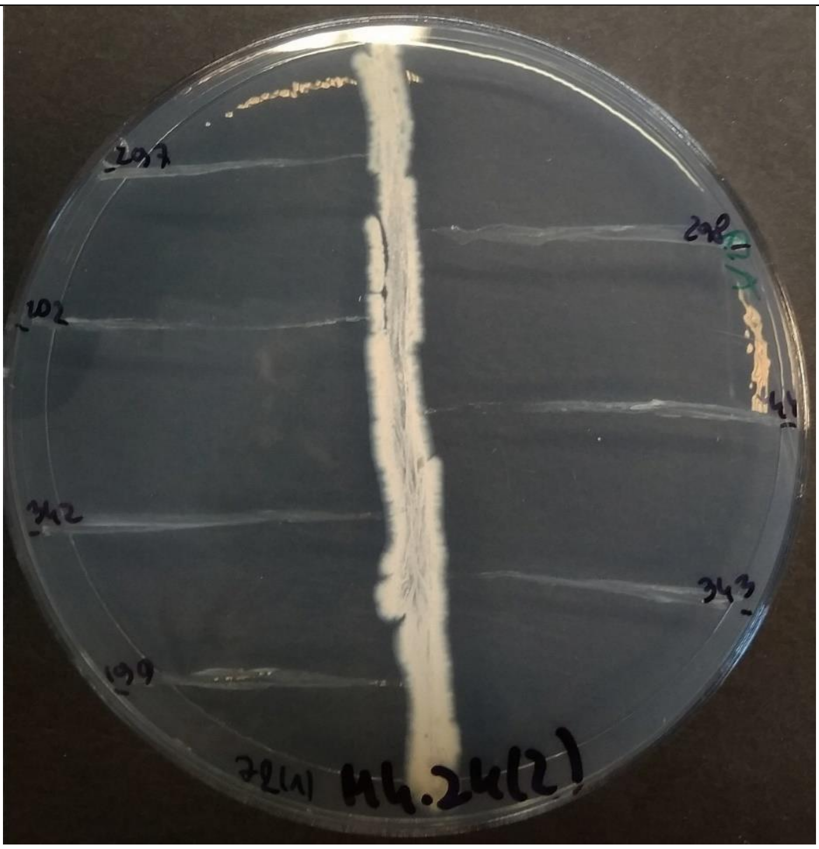<br><br>M4_24 72h I	*Staphylococcus aureus* MRSA 298 (Medical University of Gdańsk)<br><br>*Staphylococcus aureus* MRSA 44 (Medical University of Gdańsk)<br><br>*Staphylococcus aureus* MRSA 343 (Medical University of Gdańsk)
Staphylococcus aureus MRSA 352 (Medical University of Gdańsk)<br><br>*Staphylococcus aureus* MRSA 116 (Medical University of Gdańsk)<br><br>*Staphylococcus aureus* MRSA 115 (Medical University of Gdańsk)<br><br>*Staphylococcus aureus* MRSA 122 (Medical University of Gdańsk)	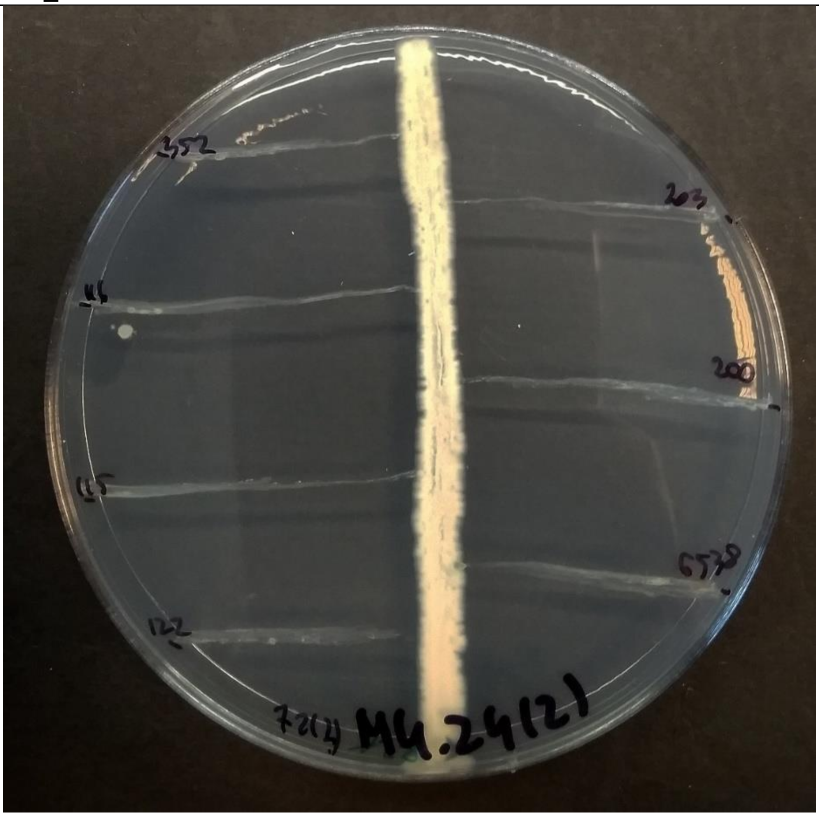<br><br>M4_24 72h II	*Staphylococcus aureus* MRSA 203 (Medical University of Gdańsk)<br><br>*Staphylococcus aureus* MRSA 200 (Medical University of Gdańsk)<br><br>*Staphylococcus aureus* MRSA ATCC 6538 (Medical University of Gdańsk)

Staphylococcus aureus MRSA 271 (Medical University of Gdańsk)<br><br>*Staphylococcus aureus* MRSA 108 (Medical University of Gdańsk)<br><br>*Staphylococcus aureus* MRSA 124 (Medical University of Gdańsk)<br><br>*Staphylococcus aureus* MRSA 149 (Medical University of Gdańsk)	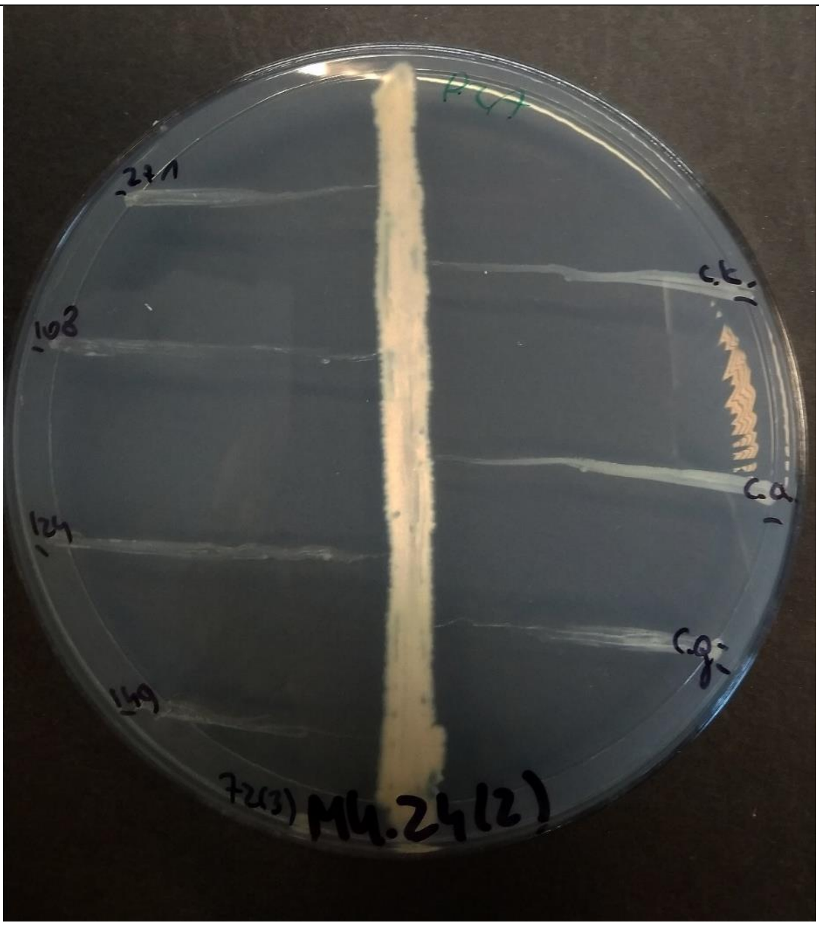<br>M4_24 72h III	-<br>-<br>-<br>(to są candida nieopisane w publikacji)
Salmonella enterica Dubin 65 (National Salmonella Center, Gdańsk, Poland)<br><br>*Salmonella enterica* Saindpaul 435 (National Salmonella Center, Gdańsk, Poland)<br><br>*Salmonella enterica* Enteritidis 1392 (National Salmonella Center, Gdańsk, Poland)<br><br>*Salmonella enterica* Newport 51 (National Salmonella Center, Gdańsk, Poland)	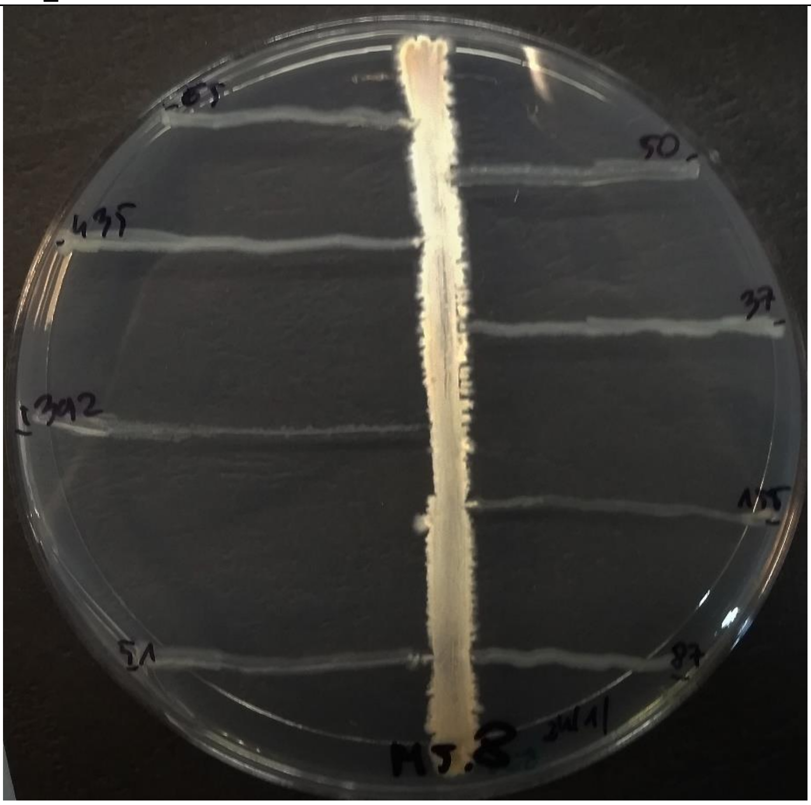<br>M5_8 24h I	*Salmonella enterica* Newport 50 (National Salmonella Center, Gdańsk, Poland)<br><br>*Salmonella enterica* Cholerasuis 37 (National Salmonella Center, Gdańsk, Poland)<br><br>*Salmonella enterica* Infantis 155 (National Salmonella Center, Gdańsk, Poland)<br><br>*Salmonella enterica* Seftenberg 87 (National Salmonella Center, Gdańsk, Poland)

Salmonella enterica Bovismorbificans 300 (National Salmonella Center, Gdańsk, Poland)<br><br>*Salmonella enterica* Gallinarum 74 (National Salmonella Center, Gdańsk, Poland)<br><br>*Salmonella enterica* Cholerasuis 39 (National Salmonella Center, Gdańsk, Poland)<br><br>*Salmonella enterica* Hadar 1784 (National Salmonella Center, Gdańsk, Poland)	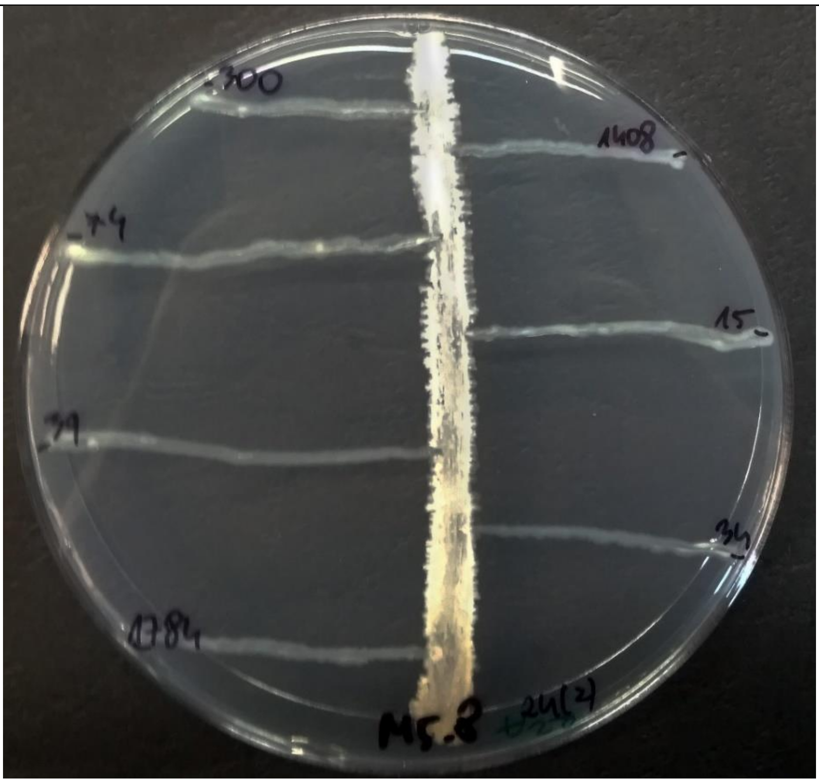<br>M5_8 24h II	*Salmonella enterica* Agona 1408 (National Salmonella Center, Gdańsk, Poland)<br><br>-<br>-
Salmonella enterica Typhimurium 13 (National Salmonella Center, Gdańsk, Poland)<br><br>*Salmonella enterica* Typhimurium 12 (National Salmonella Center, Gdańsk, Poland)<br><br>-<br><br>*Salmonella enterica* Cholerasuis 1439 (National Salmonella Center, Gdańsk, Poland)	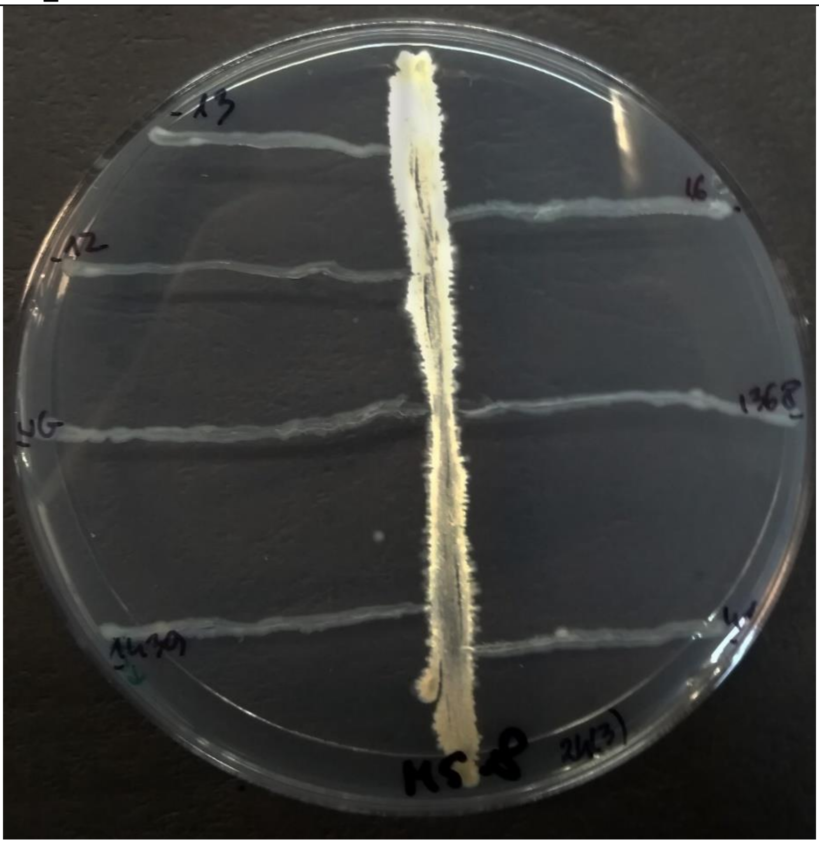<br>M5_8 24h III	*Salmonella enterica* Heidelberg 16 (National Salmonella Center, Gdańsk, Poland)<br><br>*Salmonella enterica* Kentucky 1368 (National Salmonella Center, Gdańsk, Poland)<br><br>*Salmonella enterica* Virchow 41 (National Salmonella Center, Gdańsk, Poland)

Salmonella enterica Enteritidis 64 (National Salmonella Center, Gdańsk, Poland)<br><br>-<br>-<br>-	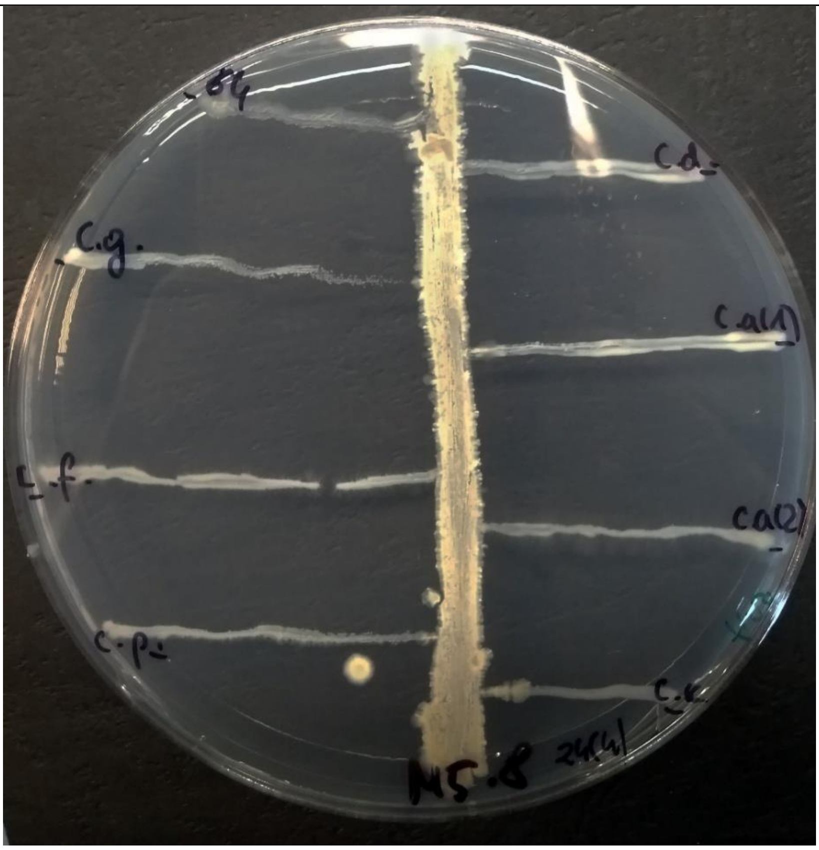<br>M5_8 24h IV	-<br>-<br>-<br>-
Salmonella enterica Dubin 65 (National Salmonella Center, Gdańsk, Poland)<br><br>*Salmonella enterica* Saindpaul 435 (National Salmonella Center, Gdańsk, Poland)<br><br>*Salmonella enterica* Enteritidis 1392 (National Salmonella Center, Gdańsk, Poland)<br><br>*Salmonella enterica* Newport 51 (National Salmonella Center, Gdańsk, Poland)	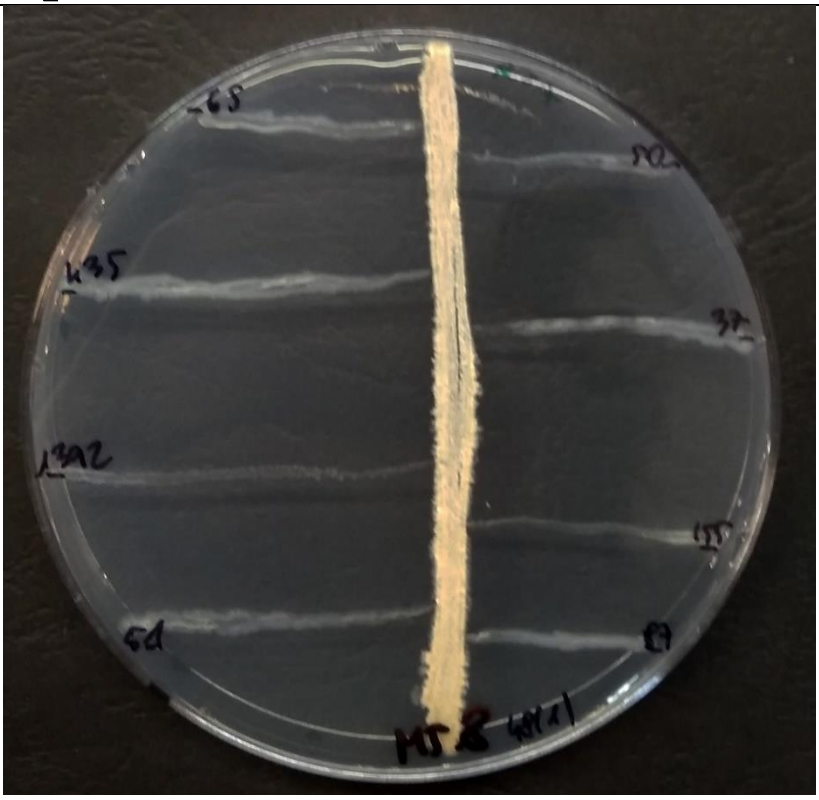<br>M5_8 48h I	*Salmonella enterica* Newport 50 (National Salmonella Center, Gdańsk, Poland)<br><br>*Salmonella enterica* Cholerasuis 37 (National Salmonella Center, Gdańsk, Poland)<br><br>*Salmonella enterica* Infantis 155 (National Salmonella Center, Gdańsk, Poland)<br><br>*Salmonella enterica* Seftenberg 87 (National Salmonella Center, Gdańsk, Poland)

Salmonella enterica Bovismorbificans 300 (National Salmonella Center, Gdańsk, Poland)<br><br>*Salmonella enterica* Gallinarum 74 (National Salmonella Center, Gdańsk, Poland)<br><br>*Salmonella enterica* Cholerasuis 39 (National Salmonella Center, Gdańsk, Poland)<br><br>*Salmonella enterica* Hadar 1784 (National Salmonella Center, Gdańsk, Poland)	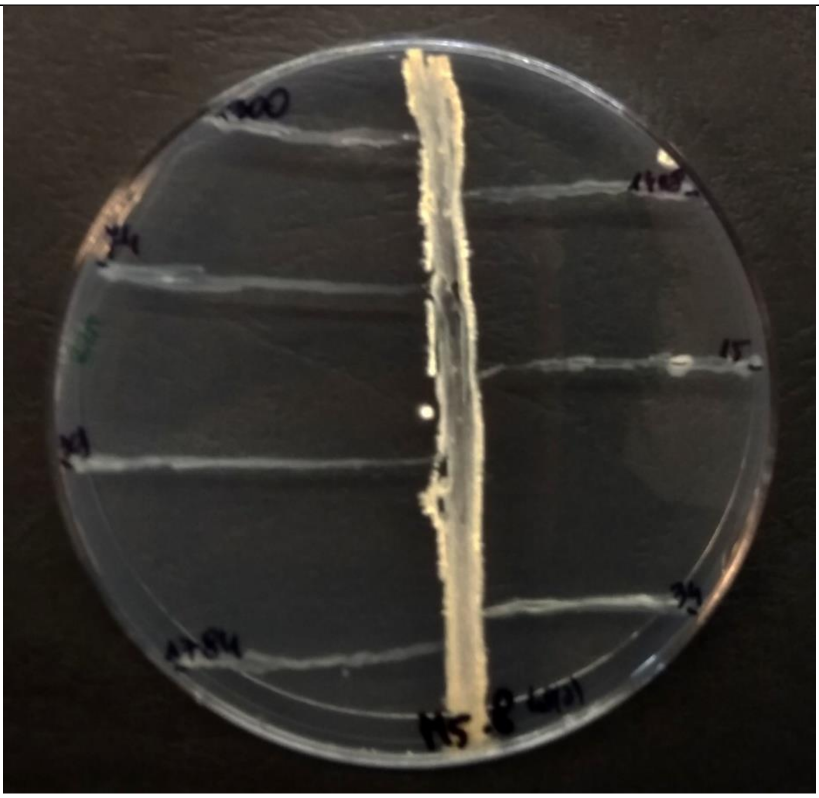<br>M5_8 48h II	*Salmonella enterica* Agona 1408 (National Salmonella Center, Gdańsk, Poland)<br><br>-<br>-
Salmonella enterica Typhimurium 13 (National Salmonella Center, Gdańsk, Poland)<br><br>*Salmonella enterica* Typhimurium 12 (National Salmonella Center, Gdańsk, Poland)<br><br>-<br><br>*Salmonella enterica* Cholerasuis 1439 (National Salmonella Center, Gdańsk, Poland)	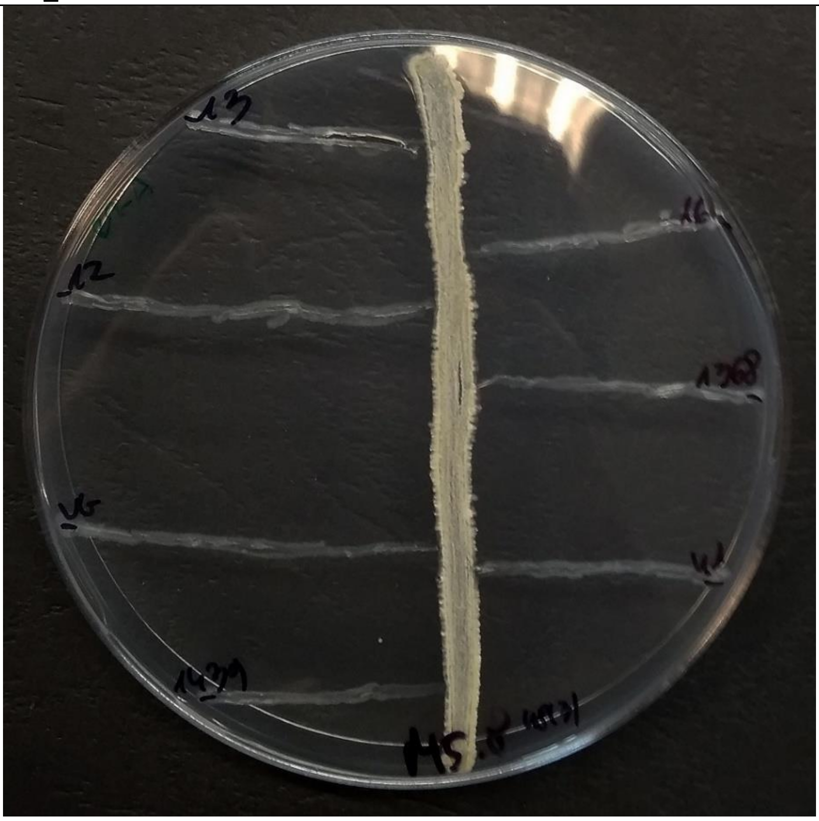<br>M5_8 48h III	*Salmonella enterica* Heidelberg 16 (National Salmonella Center, Gdańsk, Poland)<br><br>*Salmonella enterica* Kentucky 1368 (National Salmonella Center, Gdańsk, Poland)<br><br>*Salmonella enterica* Virchow 41 (National Salmonella Center, Gdańsk, Poland)

Salmonella enterica Enteritidis 64 (National Salmonella Center, Gdańsk, Poland)<br><br>-<br>-<br>-	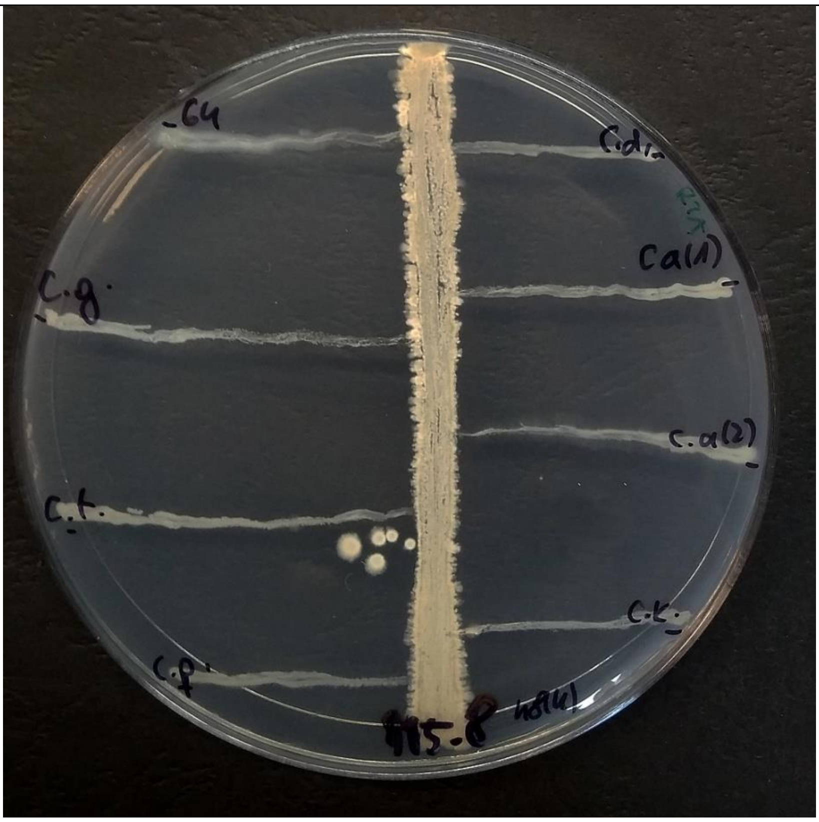<br>M5_8 48h IV	-<br>-<br>-<br>-
Salmonella enterica Dubin 65 (National Salmonella Center, Gdańsk, Poland)<br><br>*Salmonella enterica* Saindpaul 435 (National Salmonella Center, Gdańsk, Poland)<br><br>*Salmonella enterica* Enteritidis 1392 (National Salmonella Center, Gdańsk, Poland)<br><br>*Salmonella enterica* Newport 51 (National Salmonella Center, Gdańsk, Poland)	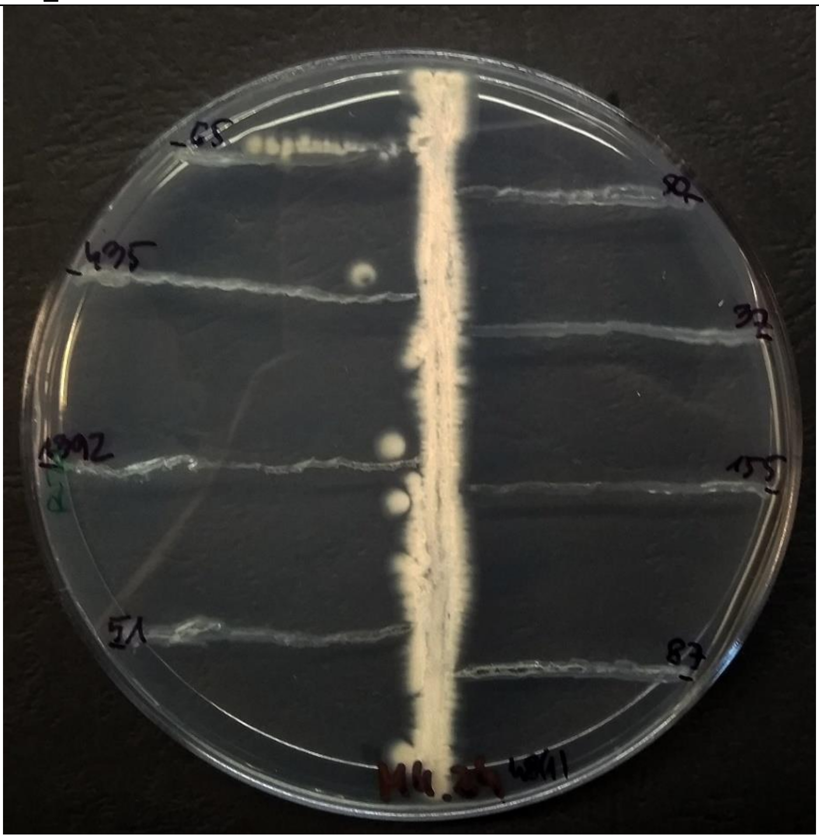<br>M4_24 48h I	*Salmonella enterica* Newport 50 (National Salmonella Center, Gdańsk, Poland)<br><br>*Salmonella enterica* Cholerasuis 37 (National Salmonella Center, Gdańsk, Poland)<br><br>*Salmonella enterica* Infantis 155 (National Salmonella Center, Gdańsk, Poland)<br><br>*Salmonella enterica* Seftenberg 87 (National Salmonella Center, Gdańsk, Poland)

Salmonella enterica Bovismorbificans 300 (National Salmonella Center, Gdańsk, Poland)<br><br>*Salmonella enterica* Gallinarum 74 (National Salmonella Center, Gdańsk, Poland)<br><br>*Salmonella enterica* Cholerasuis 39 (National Salmonella Center, Gdańsk, Poland)<br><br>*Salmonella enterica* Hadar 1784 (National Salmonella Center, Gdańsk, Poland)	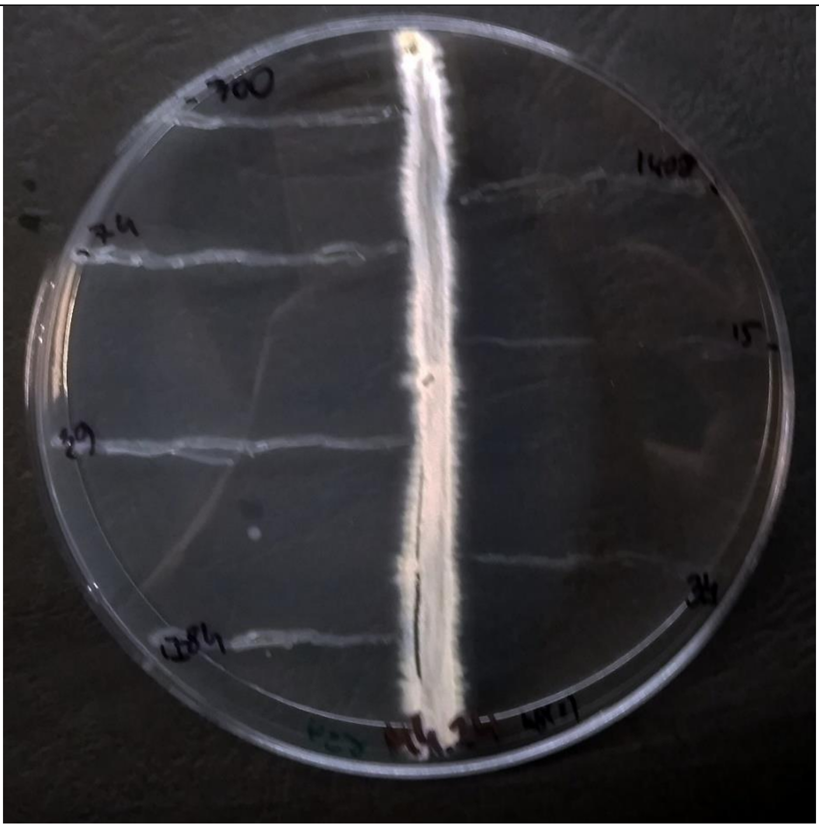<br>M4_24 48h II	*Salmonella enterica* Agona 1408 (National Salmonella Center, Gdańsk, Poland)<br><br>-<br>-
Salmonella enterica Typhimurium 13 (National Salmonella Center, Gdańsk, Poland)<br><br>*Salmonella enterica* Typhimurium 12 (National Salmonella Center, Gdańsk, Poland)<br><br>-<br><br>*Salmonella enterica* Cholerasuis 1439 (National Salmonella Center, Gdańsk, Poland)	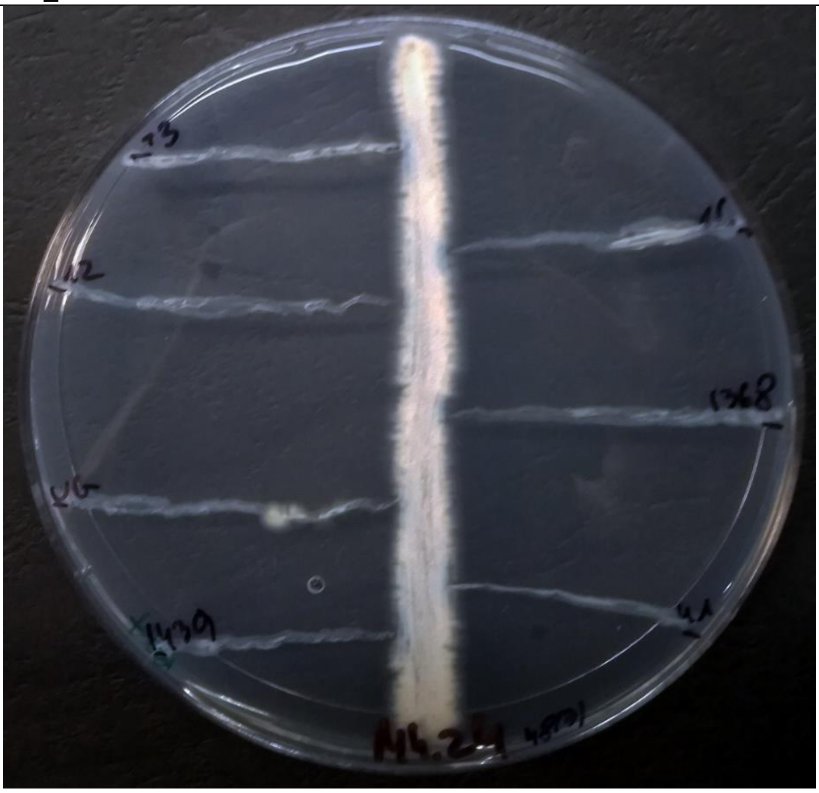<br>M4_24 48h III	*Salmonella enterica* Heidelberg 16 (National Salmonella Center, Gdańsk, Poland)<br><br>*Salmonella enterica* Kentucky 1368 (National Salmonella Center, Gdańsk, Poland)<br><br>*Salmonella enterica* Virchow 41 (National Salmonella Center, Gdańsk, Poland)

Salmonella enterica Enteritidis 64 (National Salmonella Center, Gdańsk, Poland)<br><br>-<br>-<br>-	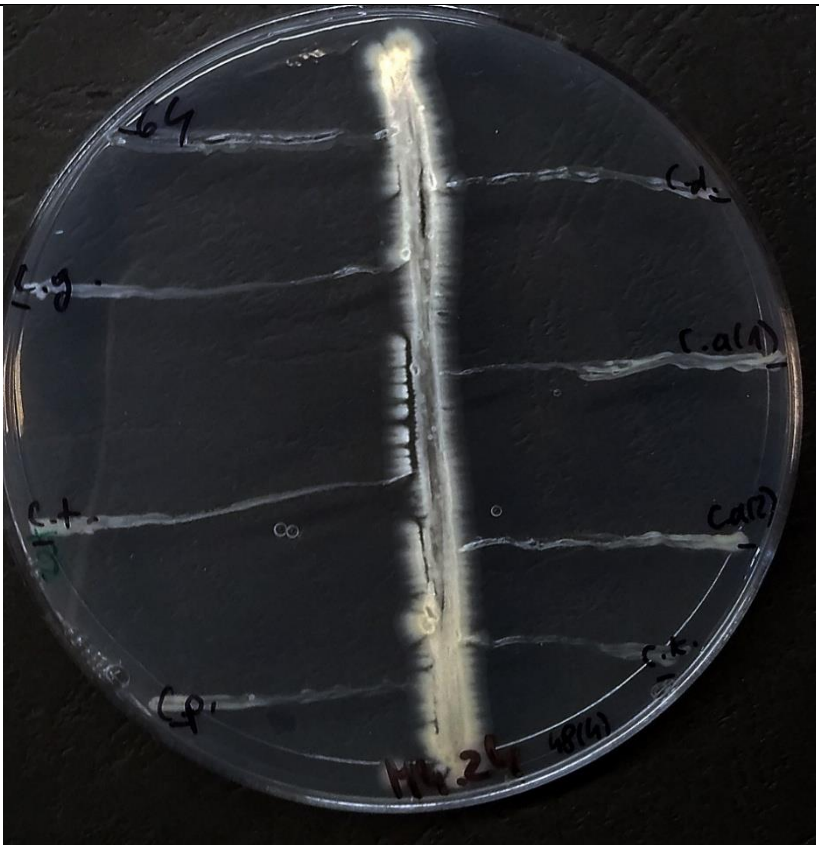<br>M4_24 48h IV	-<br>-<br>-<br>-
Salmonella enterica Dubin 65 (National Salmonella Center, Gdańsk, Poland)<br><br>*Salmonella enterica* Saindpaul 435 (National Salmonella Center, Gdańsk, Poland)<br><br>*Salmonella enterica* Enteritidis 1392 (National Salmonella Center, Gdańsk, Poland)<br><br>*Salmonella enterica* Newport 51 (National Salmonella Center, Gdańsk, Poland)	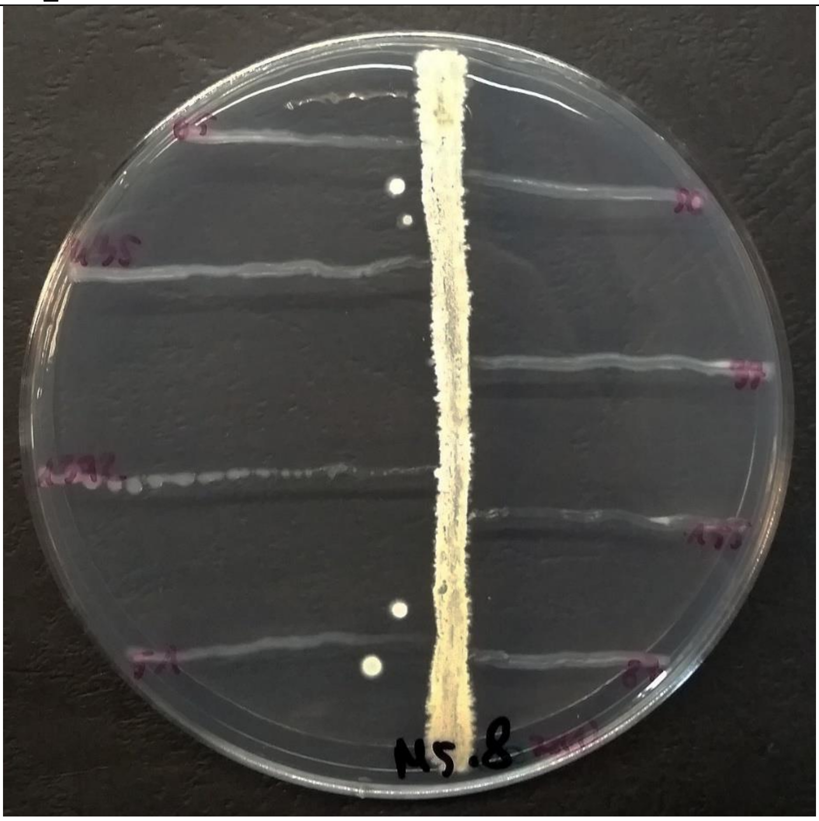<br>M5_8 72h I	*Salmonella enterica* Newport 50 (National Salmonella Center, Gdańsk, Poland)<br><br>*Salmonella enterica* Cholerasuis 37 (National Salmonella Center, Gdańsk, Poland)<br><br>*Salmonella enterica* Infantis 155 (National Salmonella Center, Gdańsk, Poland)<br><br>*Salmonella enterica* Seftenberg 87 (National Salmonella Center, Gdańsk, Poland)

Salmonella enterica Bovismorbificans 300 (National Salmonella Center, Gdańsk, Poland)<br><br>*Salmonella enterica* Gallinarum 74 (National Salmonella Center, Gdańsk, Poland)<br><br>*Salmonella enterica* Cholerasuis 39 (National Salmonella Center, Gdańsk, Poland)<br><br>*Salmonella enterica* Hadar 1784 (National Salmonella Center, Gdańsk, Poland)	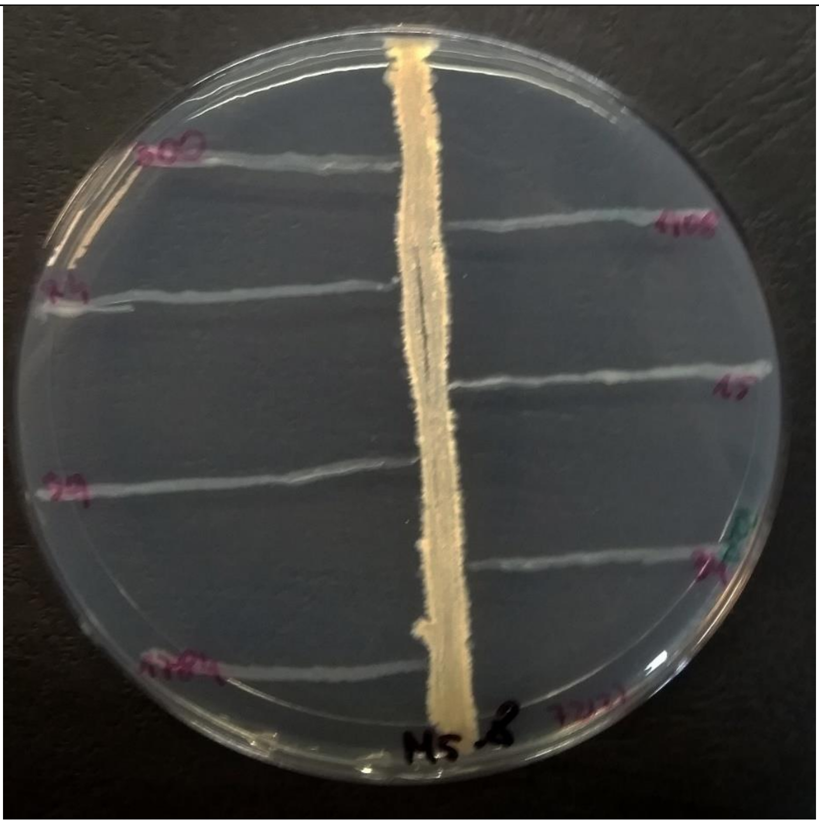<br>M5_8 72h II	*Salmonella enterica* Agona 1408 (National Salmonella Center, Gdańsk, Poland)<br><br>-<br>-
Salmonella enterica Typhimurium 13 (National Salmonella Center, Gdańsk, Poland)<br><br>*Salmonella enterica* Typhimurium 12 (National Salmonella Center, Gdańsk, Poland)<br><br>-<br><br>*Salmonella enterica* Cholerasuis 1439 (National Salmonella Center, Gdańsk, Poland)	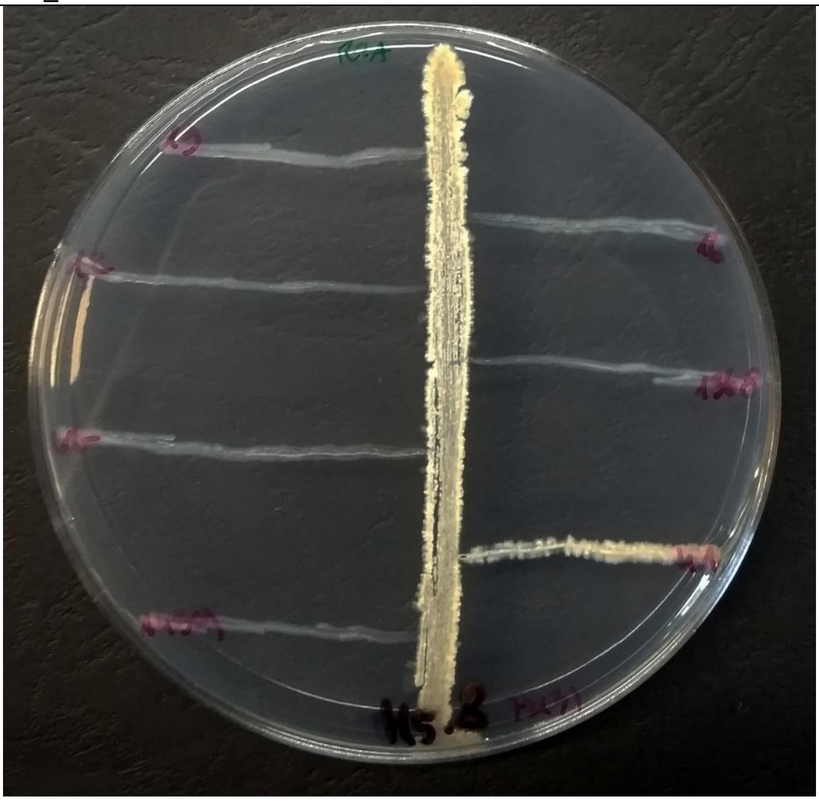<br>M5_8 72h III	*Salmonella enterica* Heidelberg 16 (National Salmonella Center, Gdańsk, Poland)<br><br>*Salmonella enterica* Kentucky 1368 (National Salmonella Center, Gdańsk, Poland)<br><br>*Salmonella enterica* Virchow 41 (National Salmonella Center, Gdańsk, Poland)

<table>
<tr><td>Salmonella enterica Enteritidis 64 (National Salmonella Center, Gdańsk, Poland)

-
-
-</td><td>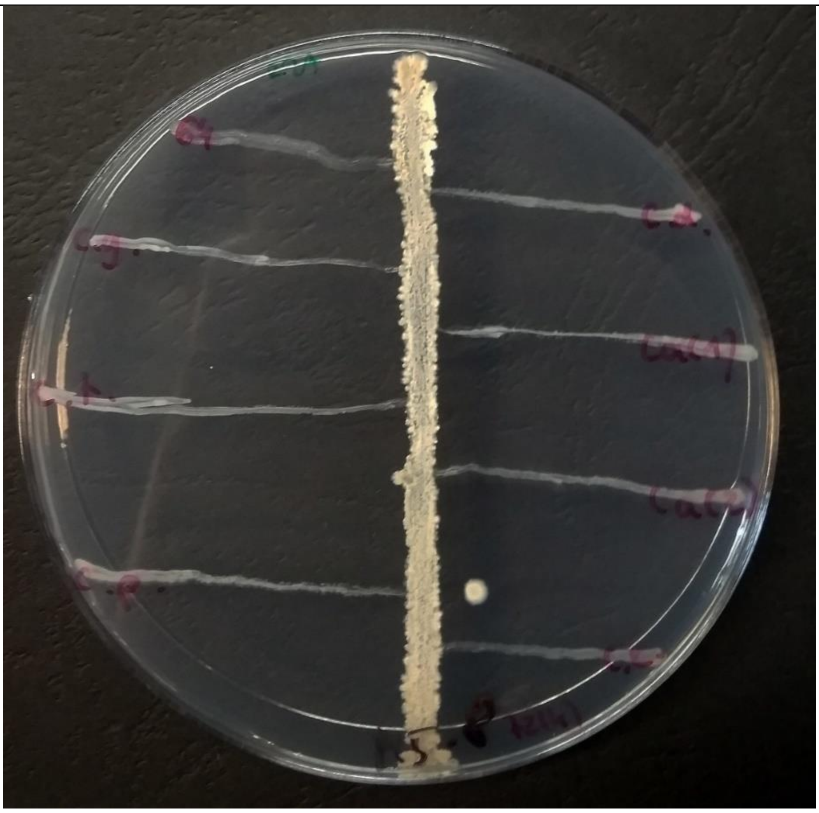
M5_8 72h IV</td><td>-
-
-
-</td></tr>
<tr><td>Salmonella enterica Dubin 65 (National Salmonella Center, Gdańsk, Poland)

Salmonella enterica Saindpaul 435 (National Salmonella Center, Gdańsk, Poland)

Salmonella enterica Enteritidis 1392 (National Salmonella Center, Gdańsk, Poland)

Salmonella enterica Newport 51 (National Salmonella Center, Gdańsk, Poland)</td><td>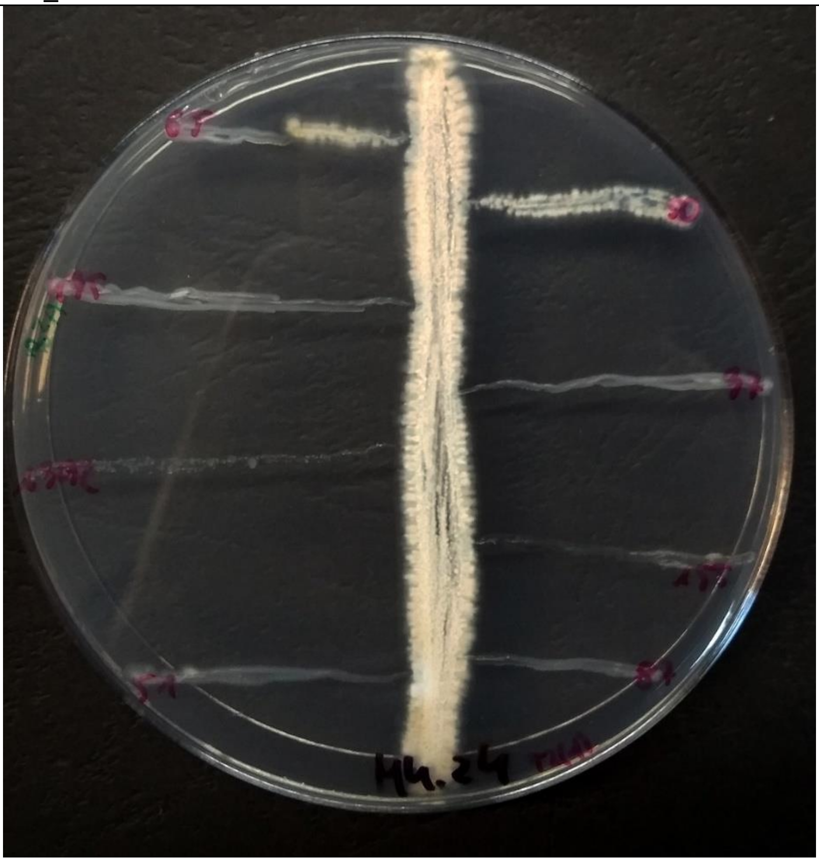
M4_24 72h I</td><td>Salmonella enterica Newport 50 (National Salmonella Center, Gdańsk, Poland)

Salmonella enterica Cholerasuis 37 (National Salmonella Center, Gdańsk, Poland)

Salmonella enterica Infantis 155 (National Salmonella Center, Gdańsk, Poland)

Salmonella enterica Seftenberg 87 (National Salmonella Center, Gdańsk, Poland)</td></tr>
</table>

Salmonella enterica Bovismorbificans 300 (National Salmonella Center, Gdańsk, Poland)<br><br>*Salmonella enterica* Gallinarum 74 (National Salmonella Center, Gdańsk, Poland)<br><br>*Salmonella enterica* Cholerasuis 39 (National Salmonella Center, Gdańsk, Poland)<br><br>*Salmonella enterica* Hadar 1784 (National Salmonella Center, Gdańsk, Poland)	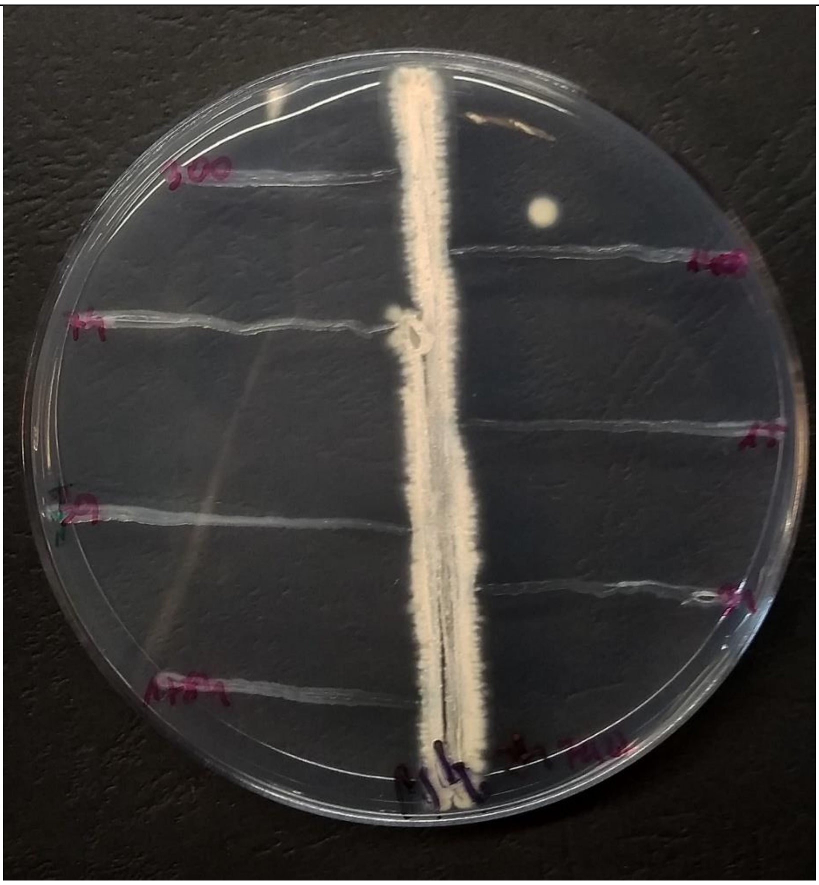<br>M4_24 72h II	*Salmonella enterica* Agona 1408 (National Salmonella Center, Gdańsk, Poland)<br><br>-<br>-
Salmonella enterica Typhimurium 13 (National Salmonella Center, Gdańsk, Poland)<br><br>*Salmonella enterica* Typhimurium 12 (National Salmonella Center, Gdańsk, Poland)<br><br>-<br><br>*Salmonella enterica* Cholerasuis 1439 (National Salmonella Center, Gdańsk, Poland)	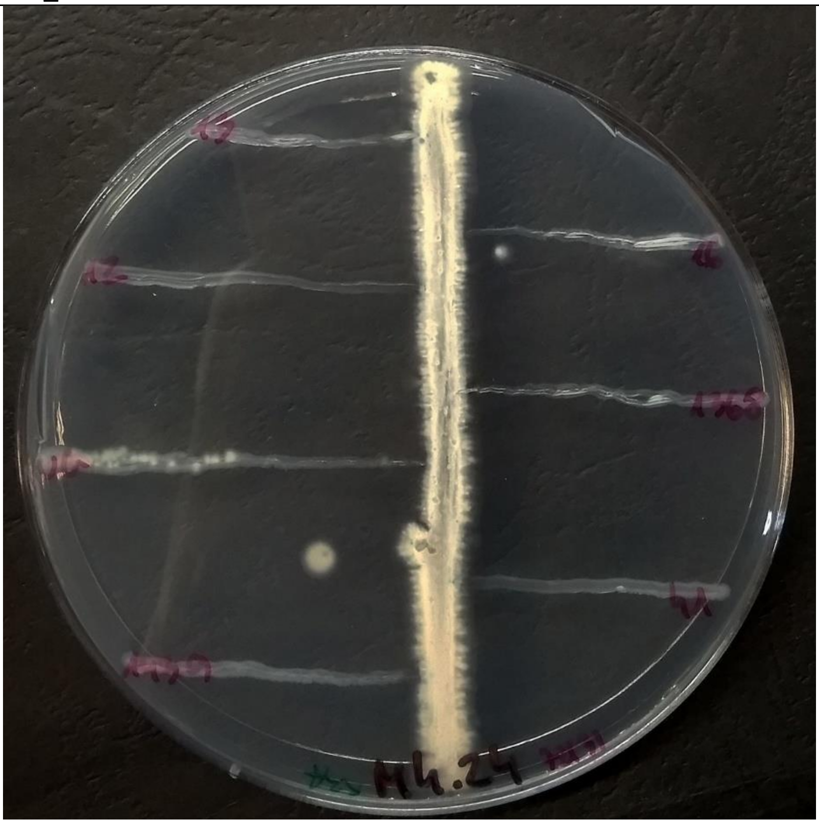<br>M4_24 72h III	*Salmonella enterica* Heidelberg 16 (National Salmonella Center, Gdańsk, Poland)<br><br>*Salmonella enterica* Kentucky 1368 (National Salmonella Center, Gdańsk, Poland)<br><br>*Salmonella enterica* Virchow 41 (National Salmonella Center, Gdańsk, Poland)

Salmonella enterica Enteritidis 64 (National Salmonella Center, Gdańsk, Poland)<br><br>-<br>-<br>-	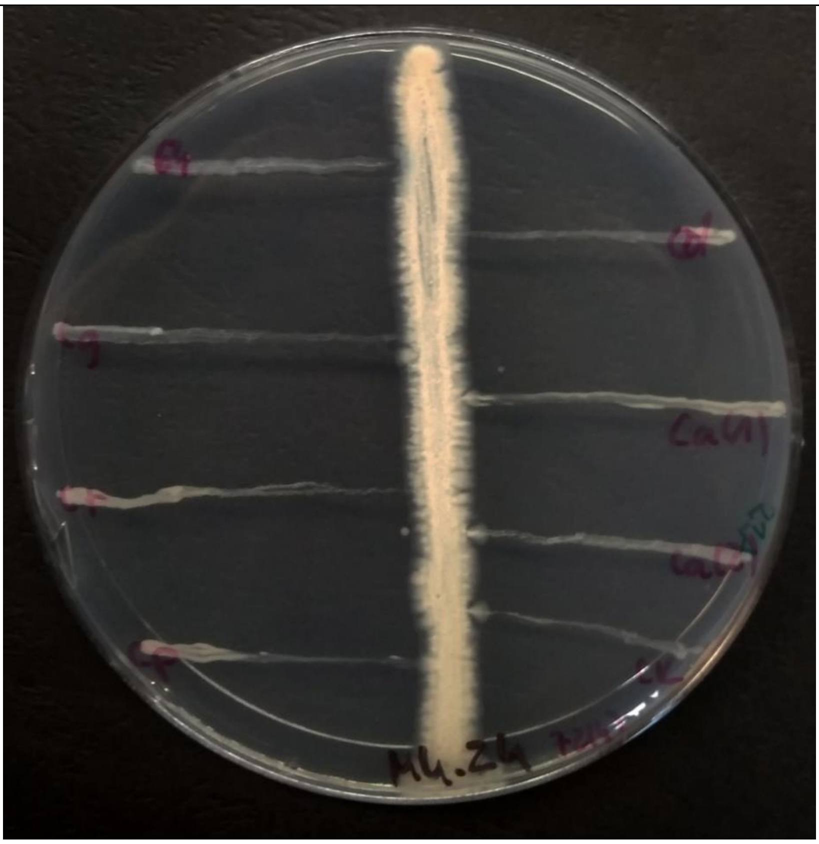<br>M4_24 72h IV	-<br>-<br>-<br>-

Figure S1. Antibacterial and antifungal activities of newly isolated *Streptomyces* strains M4_24 and M5_8, as revealed by the streak-test. Name of the *Streptomyces* strain and time of incubation are shown at the bottom of each panel. Names of streaked bacterial and fungal strains (and their origin (collection)) are provided in left and right panels.
